# Shedding light on the Early Pleistocene of TD6 (Gran Dolina, Atapuerca, Spain): The technological sequence and occupational inferences

**DOI:** 10.1371/journal.pone.0190889

**Published:** 2018-01-25

**Authors:** Marina Mosquera, Andreu Ollé, Xose Pedro Rodríguez-Álvarez, Eudald Carbonell

**Affiliations:** 1 Àrea de Prehistòria, Universitat Rovira i Virgili (URV), Avinguda de Catalunya 35, Tarragona, Spain; 2 Institut Català de Paleoecologia Humana i Evolució Social (IPHES-CERCA), Zona Educacional 4, Campus Sescelades URV (Edifici W3), Tarragona, Spain; Max Planck Institute for the Science of Human History, GERMANY

## Abstract

This paper aims to update the information available on the lithic assemblage from the entire sequence of TD6 now that the most recent excavations have been completed, and to explore possible changes in both occupational patterns and technological strategies evidenced in the unit. This is the first study to analyse the entire TD6 sequence, including subunits TD6.3 and TD6.1, which have never been studied, along with the better-known TD6.2 *Homo antecessor*-bearing subunit. We also present an analysis of several lithic refits found in TD6, as well as certain technical features that may help characterise the hominin occupations. The archaeo-palaeontological record from TD6 consists of 9,452 faunal remains, 443 coprolites, 1,046 lithic pieces, 170 hominin remains and 91 *Celtis* seeds. The characteristics of this record seem to indicate two main stages of occupation. In the oldest subunit, TD6.3, the lithic assemblage points to the light and limited hominin occupation of the cave, which does, however, grow over the course of the level. In contrast, the lithic assemblages from TD6.2 and TD6.1 are rich and varied, which may reflect Gran Dolina cave’s establishment as a landmark in the region. Despite the occupational differences between the lowermost subunit and the rest of the deposit, technologically the TD6 lithic assemblage is extremely homogeneous throughout. In addition, the composition and spatial distribution of the 12 groups of lithic refits found in unit TD6, as well as the *in situ* nature of the assemblage demonstrate the high degree of preservation at the site. This may help clarify the nature of the Early Pleistocene hominin occupations of TD6, and raise reasonable doubt about the latest interpretations that support the *ex situ* character of the assemblage as a whole.

## Introduction

TD6 is one of the primary lithostratigraphic units of the Gran Dolina site, located in the Sierra de Atapuerca near the city of Burgos (northern Spain). The Sierra de Atapuerca is an anticlinal ridge (1085 m a.s.l.) mainly formed by Late Cretaceous limestones and dolostones [1, 2, 3]. This area of about 25 km^2^ houses several archaeo-palaeontological sites, ranging from Early Pleistocene to Holocene in age [4, 5]. Of those sites, Gran Dolina is of particular importance. This cave has a keyhole profile [6, 7] infilled with 25 m of Early and Middle Pleistocene sediments [2].

To date, 12 lithostratigraphic units have been identified at Gran Dolina [2:73–74 for description]. TD6 is a three metre-thick unit divided into three subunits, from oldest to youngest: TD6.3, TD6.2 and TD6.1. TD6.3 comprises at least six debris flow events from a northwest entry point with distal *facies* towards the southeast, which is the opposite direction to the underlying TD5 sediment. The sedimentary processes in TD6.2 and TD6.1 are different from those of TD6.3. In TD6.1 and TD6.2, channel processes dominate, with thicker gravel layers than in previous units, probably deposited when the regional base level corresponded to fluvial terrace T4 (+62 m) of the Arlanzón River [8]. Towards the south, channel *facies* change laterally to silts and muds described as floodplain *facies* [9]. This section of TD6.2 has been defined as the “Aurora archaeostratigraphic set” (AAS) [10], and contains human fossils, stone tools, and faunal remains. TD6 ends with a red decantation *facies* indicating slow sedimentation and a major temporal hiatus between TD6 and TD7 [9]. TD7 is about 1 m thick, composed of laminated silts at the base and cemented gravels at the top. A palaeomagnetic polarity reversal has been reported at the top of TD7, attributed to the Matuyama-Brunhes boundary, and indicating the transition between the Early and Middle Pleistocene at Gran Dolina [11].

Unit TD6 has been the subject of several studies, primarily focusing on hominins [10, 12–29], archaeological stratigraphy [30], cannibalism [31–34], the sedimentary sequence [1, 35, 36], geochronology [11, 37–42], paleoecology and palaeontology [43–50], the paleoenvironment [51–53], archaeozoology [54–57], and the lithic assemblage [58–64].

However, most of these studies involved partial assemblages, primarily because the excavations in the southern section of Gran Dolina have only recently been completed (2011 field season). In addition, most of these publications focused on TD6.2, the hominin-bearing subunit, and disregarded subunits TD6.3 and TD6.1, which also contain data of archaeo-paleontological interest.

TD6.2 was first suggested to be a base-camp [58, 65], but recent geological studies concluded that the sediments are from different *facies* and inputs, and interpreted the site with the archaeo-paleontological remains as being in a secondary position [1, 9].

## Materials and methods

**Fig 1:**
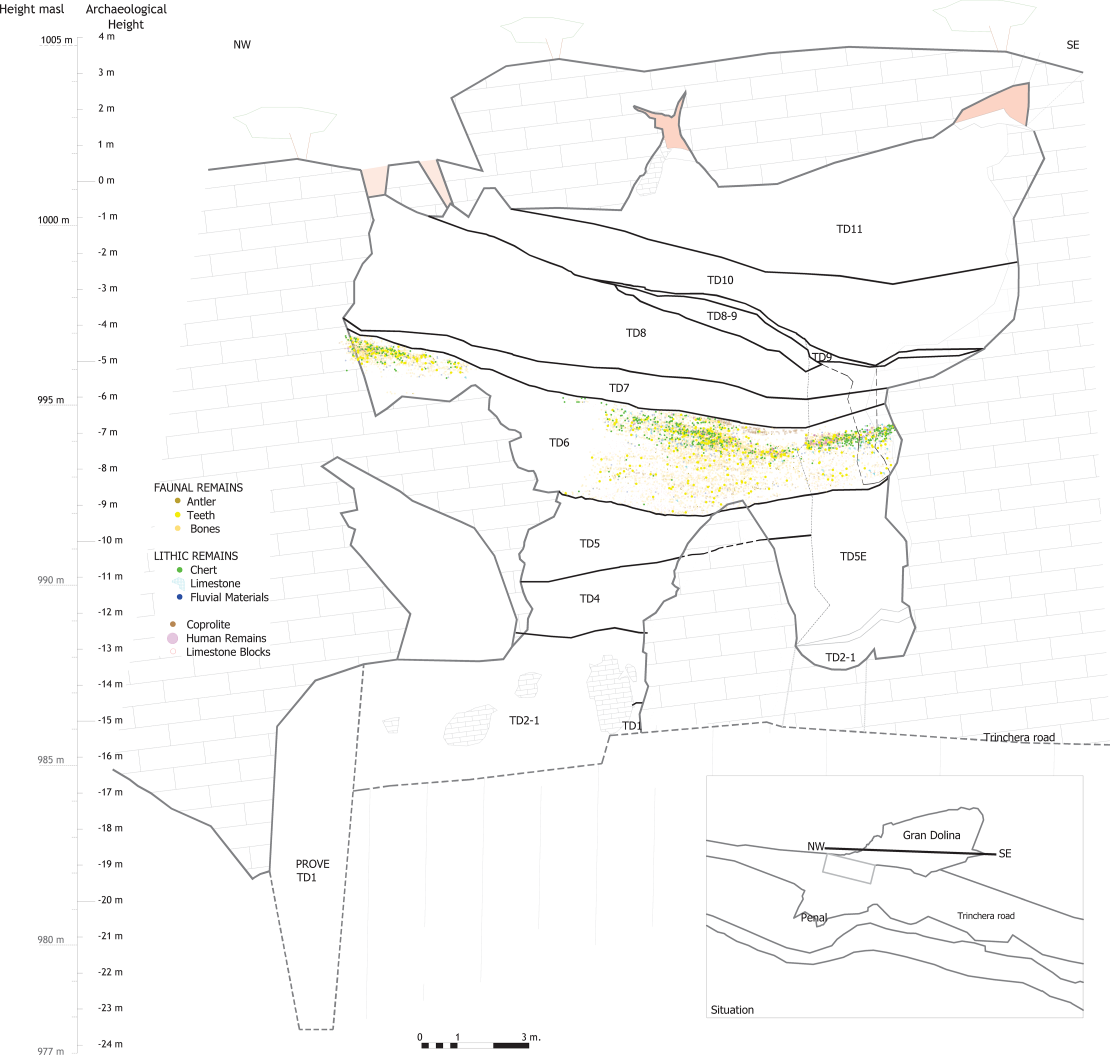
Stratigraphic section of the Gran Dolina site and vertical distribution of the TD6 archaeo-palontological remains excavated to date (R. Pérez).

**Fig 2:**
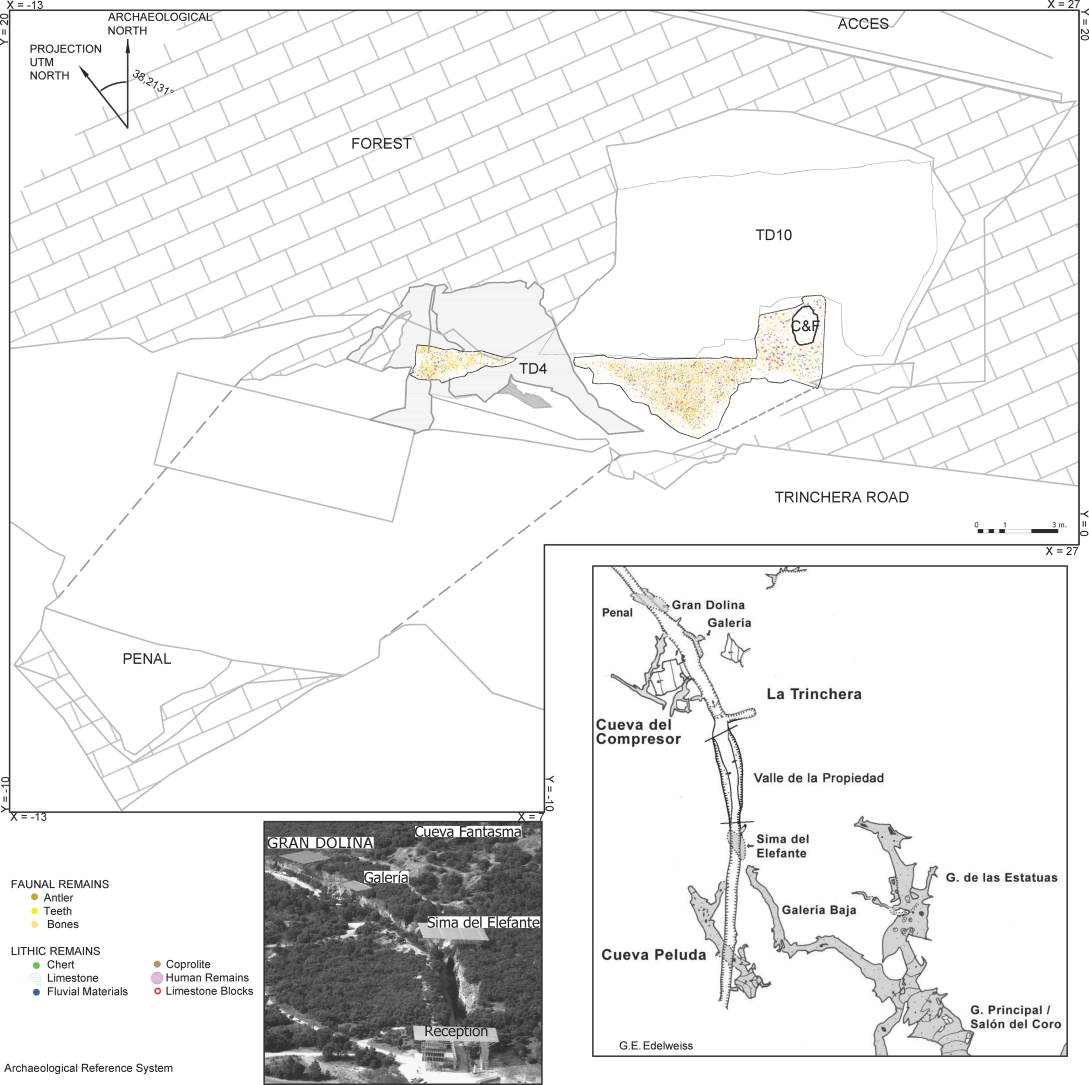
Horizontal map of the Gran Dolina site, with the spatial distribution of the TD6 archaeo-paleontological remains excavated to date (R. Pérez).

The study area is approximately 20m^2^ (Figs 1 and 2), and corresponds to the southern section of Gran Dolina (according to archaeological north), which was excavated in two stages: the eastern area (the Test Pit), excavated between 1994 and 1997, and the western (Torreón) and central areas, excavated between 2003 and 2011.

The complete archaeo-paleontological collection from TD6 consists of 10,880 three-dimensionally recorded remains, of which 9,452 are faunal (286 antler fragments, 445 teeth, 8,721 bones, and 443 coprolites), 91 are *Celtis* seeds [66], 1,046 are lithic pieces, and 170 are hominin remains (26 teeth and 144 bones). All necessary permits were obtained for this study, which complied with all regulations from Dirección General de Patrimonio-Junta de Castilla y León. Lithic materials described in this study are partly deposited in the Museo de Burgos (Spain) (until 2005 field season), and Institut de Paleoecologia Humana I Evolució Social (IPHES) (Tarragona, Spain) (2006–2011).

The archaeo-paleontological remains are distributed over three subunits (TD6.1, TD6.2 and TD6.3 from top to bottom), and several layers. Some of these layers were extremely difficult to distinguish over the entire area during the excavations due to various lateral sedimentary discontinuities [1, 9]. In addition, the sedimentary sequence of the eastern area (Test Pit) is compressed, and does not have the same sedimentary characteristics as the western and central areas.

Furthermore, it is important to bear in mind that the excavated area is not continuous, making this correlation even more difficult. Unfortunately, a recent work on the TD6 stratigraphy [1] only analyses, describes and numbers the layers of the central area (squares 9 to 15), leaving aside the western (squares 3 to 8) and the eastern zones (squares 16 to 18) of Gran Dolina, where a high number of archaeo-paleontological items have been recovered, including many hominin remains. In addition, this geological study describes the layers in the section, but not on the excavation surface. Finally, these layers are not correlated in the publication with the archaeo-paleontological material or the layers identified during the excavation. In summary, there is no way of correlating the sedimentary layers proposed in Campaña et al. with the archaeo-paleontological material and the layers identified during excavation. Therefore, this study correlates the material from the two abovementioned excavation phases, by systematically ascribing the entire set of archaeo-paleontological materials to the profiles [1, 2], the gaps with no remains, and the limestone block beds.

**Fig 3:**
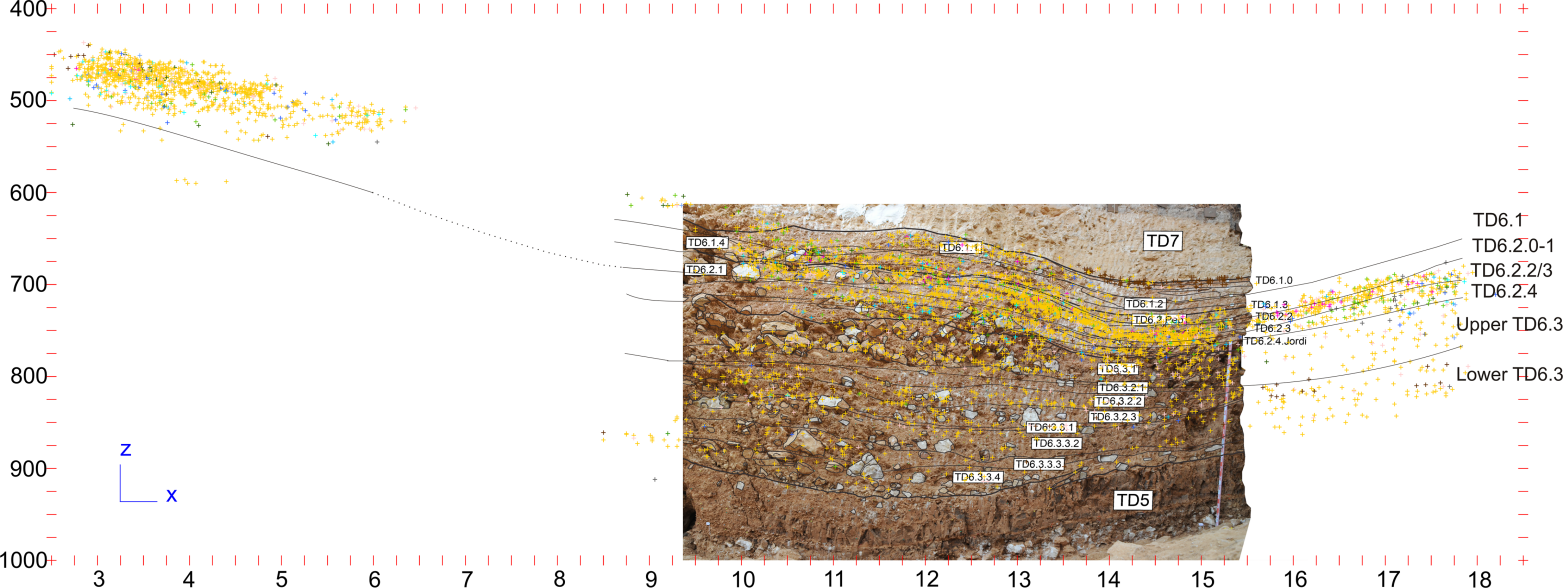
Subunits of TD6 in Raw G, superimposed to the stratigraphic section described by Campaña et al. 2016, in the contact of Rows G and H. Yellow: bones; light pink: antler; light yellow: teeth; brown: coprolites; pink: hominin remains; blue: lithic remains in fluvial materials (sandstone, quartz, quartzite and quartzarenite); green: lithic remains in Neogene and Cretaceous chert (georeferenced image provided by I. Campaña).

Eventually, we managed to correlate the different layers as follows (Table 1) (Fig 3): subunit TD6.1, the youngest, includes the “Techo” (Top) of the western area, the main part of TD6.1 in the central area, as well as layers 32, 34 and 35 in the eastern area. Subunit TD6.2 comprises modern layers 0 to 4. Layers TD6.2.0 and TD6.2.1 correlate with eastern area layers 36 to39, which are difficult to individualise and have been grouped together in this work as TD6.2.0–1. Layers 2 and 3 of TD6.2 were difficult to distinguish in most of the modern excavation areas, so they were grouped as layer 2/3. This layer-group corresponds to eastern area layers 38–39 and 40–41, all of which have been grouped in this work as TD6.2.2/3. Layer TD6.2.4 of the western and central areas correlates with layers 40, 41, 42, 43, 44, 45, layer 40–45 and layer 38–40 of the eastern area, and all are called TD6.2.4 in this work. Finally, subunit TD6.3, the oldest subunit, is represented by layers 0 to 7 in the western and central areas, and layers 45 to 55 in the eastern area. These have been grouped as follows: western and central layers 0 to 4 correlate with eastern layers 45 to 51, and have been named Upper-TD6.3; western and central layers 5 to 7 correlate with eastern layers 52 to 55, and have been named Lower-TD6.3.

**Table 1:** Correlation between layers of TD6.

Subunit	Excavation layers West and Central areas (squares 03 to 15)			Campaña et al. 2016 (Central area, squares 09 to 15)	Excavation layers East area (Test Pit) (squares 16 to 18)	This work
TD6.1	“Techo” (Top) and TD6.1			TD6.1.0 to TD6.1.4	32, 34 and 35	TD6.1
TD6.2	0			TD6.2.Pep	36 to 39	TD6.2.0–1
	1			TD6.2.1		
	2/3; 2/3a to 2/3c	2/3	2	TD6.2.2	38–39 and 40–41	TD6.2.2/3
			3	TD6.2.3		
	4			TD6.2.4 Jordi	40 to 44, 40–45 and 38–40	TD6.2.4
TD6.3	0 to 4			TD6.3.1, TD6.3.2 (TD6.3.2.1 to 3), TD6.3.3 (TD6.3.3.1 to 4)	45 to 51	Upper-TD6.3
	5 to 7				52 to 55	Lower-TD6.3

Correlation between the two phases of excavation in the different areas of TD6, and the sedimentary sequence proposed by Campaña et al., 2016.

We considered the general archaeo-palaeontological composition of the layers, as well as the hominins that can be ascribed to each layer and their age range if available, based on previous studies [14, 17–23, 25, 26, 32, 67] (see [68] for label correlations). We analysed the technical features of each category of lithic artefacts (cores, flakes, hammerstones, retouched flakes, etc.). The technical dimensions of the items were measured according to the minimal rectangle or “box method” [69]. We used the SLA method [61] for the morpho-technical analyses.

The knapping methods represented in TD6 (see [63] for further details) are:

1. Unipolar longitudinal: a single surface is typically knapped, and flake scars run unidirectionally along the flaked surface of the blank.
2. Centripetal, unifacial or
3. Bifacial: recurrent knapping around the edge of a blank.
4. Multifacial orthogonal: based on the continuous creation of surfaces that are used both as striking and detaching platforms; the angles between these surfaces tend to be close to 90°.
5. Bipolar-on-anvil technique: because the core is supported on an anvil, each strike can result in the detached flake or the core having two opposing impact points.
6. Pebbles, broken pebbles, and fragments of pebbles have been called “percussive material”, as these were transported to the cave by hominins and most bear battering marks (macrofractures, crushing, pitting and surface abrasion) suggesting their use as hammerstones for striking or knapping.

Finally, we also analysed the presence and type of refits, as well as the distance between the refit pieces. The aim was to find additional evidence that could be used to demarcate the layers within each excavated area, and correlate the layers excavated in different stages.

### Lithic raw materials

The Sierra de Atapuerca and its surroundings presented hominins with a wide range of raw materials during the Pleistocene, all found no further than 3 km from the sites [70].

Five primary rock types have been identified archaeologically: chert (including Neogene and Cretaceous material), quartzite (including quartzarenite, orthoquartzite and metaquartzite), sandstone (including sandstone, metasandstone and schist), quartz, and limestone.

The chert is divided into two petrological groups based on its origin: either Cretaceous or Neogene. The Neogene chert is from the Late Miocene and it is found as large blocks. Irregularities in the texture and quality of this chert, from block to block or even within the same block, limit its workability (the interior tends to be coarser grained and contains abundant geodes, while the exterior has a finer and more homogeneous structure). Nevertheless, its abundance in the plains surrounding the Sierra de Atapuerca, its ready availability, and the profusion of blocks of suitable sizes, make it the predominant raw material in the archaeological record.

The Cretaceous chert is from the Turonian-Lower Santonian, and there are two sources a short distance from the Trinchera del Ferrocarril sites. The first outcrop consists of a fossiliferous chert with a microcrystalline wackestone structure, and it is found towards the SSE end of the hill, and is. Nodules are 20 cm in diameter on average. The second outcrop is located in the central area of the Sierra de Atapuerca, and offers smaller nodules rarely exceeding 10 cm in diameter. Chert from this outcrop contains fewer fossils and has a compact siliceous mudstone texture. Both varieties outcrop in easily accessible exogenous karstic formations. Their suitability for knapping differs slightly, as the smaller nodules are better than the larger pieces for this purpose.

The archaeological chert, particularly the Neogene chert but also one Cretaceous variety, typically has an altered shape with a disintegrated internal structure (see Figs 18D, 20B, 21I and 23H, for examples), which often hinders its technological and functional analyses.

In addition to chert, other materials were easily accessible on the Quaternary terraces of the Arlanzón and Vena rivers, only 1 km from the sites. These rivers obtain their Palaeozoic load from La Demanda ridge, 15 km southeast of Atapuerca. Among these fluvial materials, sandstones are particularly common (some of them quartzitic in nature; i.e., orthoquartzite and quartzarenite), as are metamorphic rocks such as metasandstone, schist, shale and, less abundantly, some strata of quartzite and infilled quartz veins. These rivers also carry high-quality metaquartzite, quartz and orthoquartzite.

For the technological study, some of the lithologies identified in these source areas have been grouped as either quartzite or sandstone, based on their mechanical properties (fragility, tenacity, etc.) and certain macroscopic textural features. All of these rocks exhibit a gradation of quartz composition, cement type, and degree of metamorphism. Thus, the tenacious rocks (compact quartzarenite), and the very tenacious lithologies (metaquartzite, orthoquartzite) have been generically labelled quartzite. Less tenacious (non-compact quartzarenite) and soft rocks (sandstone, metasandstone) have also been grouped together and generically labelled as sandstone.

Finally, the limestone originated in the karstic Cretaceous substratum of the Sierra de Atapuerca. It is likely that some of the knapped cobbles were procured from the uppermost parts of the Pico River, which erodes that *facies*.

The knapping and production properties of all these raw materials can be consulted in [71].

## Results

### TD6.3

This is the thickest subunit of TD6, and has yielded evidence of the earliest hominin occupation in TD6. Subunit TD6.3 was mainly formed by sediment gravity flows [1]. Here, TD6.3 has been divided into two stages: Lower-TD6.3 and Upper-TD6.3, based on the stratigaphic correlation, the presence of an archaeological gap between the two divisions, and some limestone block layers and a coprolite layer that seem to divide the sequence into two distinct deposits (see Fig 2).

#### Lower-TD6.3

This stage contains 1,153 faunal remains (937 bones, 49 teeth, 40 antler fragments, and 126 coprolites), and 28 lithic pieces (Table 2). There are no hominin remains.

**Table 2:** Number and type of lithic items.

Lower-TD6.3	Sand	Lime	Qrtz	Qz	Qrtza	Chert	Neog	Total
**Pebble**		1	2					**3**
**Hammerstone**	1	1	4					**6**
**Fractured pebble**		2	4		1			**7**
**Fragment of pebble**			1					**1**
**Flake**		1					2	**3**
**Fragment / chunk**		1		1			1	**3**
**Indet.**	1	1				1	2	**5**
**Total**	**2**	**6**	**11**	**1**	**1**	**1**	**5**	**28**

Indet: indeterminable pieces due to their poor preservation; Sand: sandstone; Lime: limestone; Qrtz: quartzite; Qz: Quartz; Qrtza: quartzarenite; Chert: indeterminate chert; Neog: Neogene chert.

**Fig 4:**
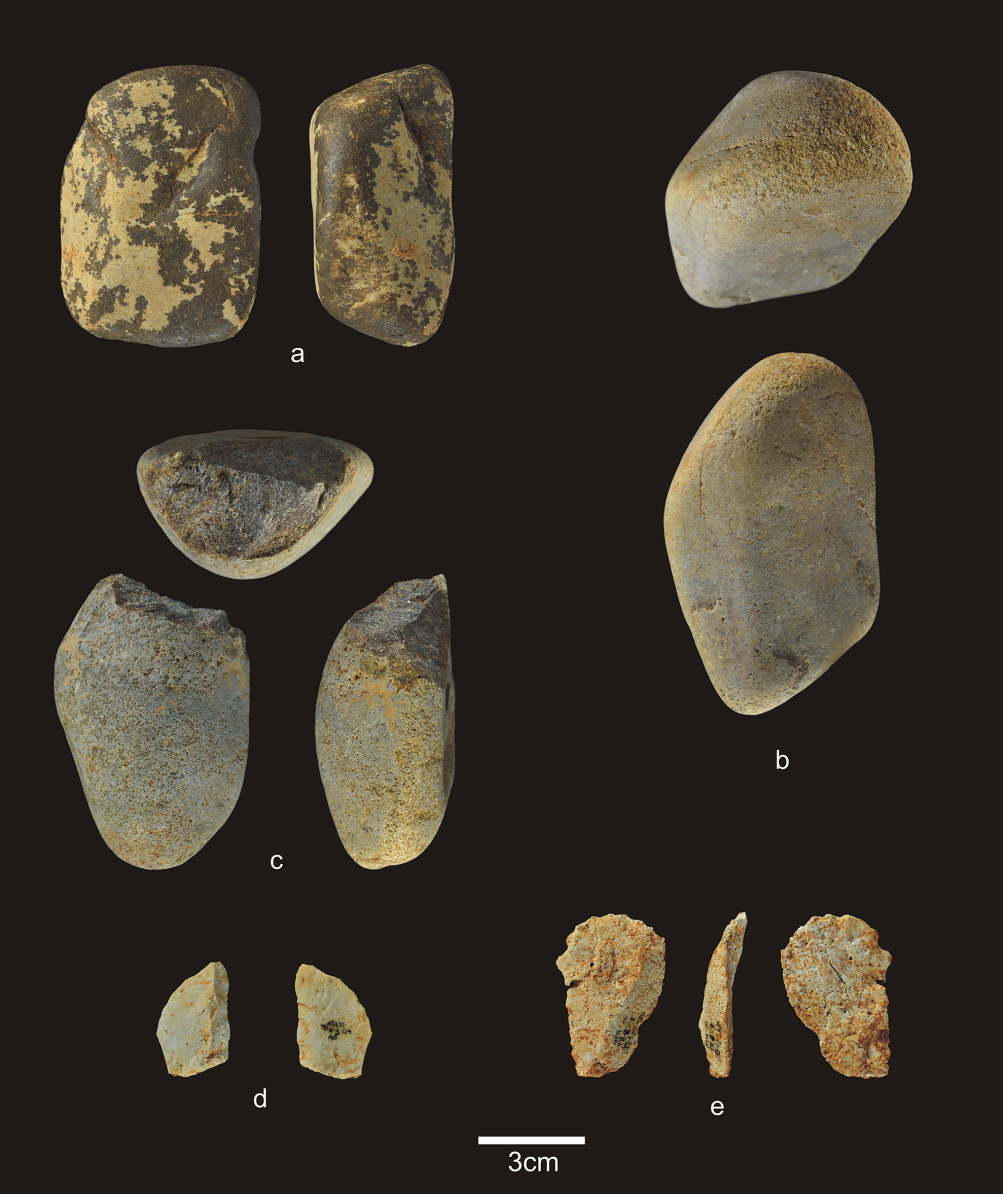
Lithic remains of Lower TD6.3. a) Ata10-E13-219, quartzite pebble; b) Ata10-G11-309, quartzite hammerstone; c) Ata10-G10-183, fractured quartzite pebble; d) Ata11-G09-40, limestone flake; e) Ata10-G12-150, Neogene chert flake.

The 28 lithic pieces consist of (Fig 4): 3 fragments, 5 indeterminable pieces, 3 simple flakes and 17 complete or fractured pebbles.

Only three complete pebbles show no traces of percussion. This means that a high proportion of pebbles (82%) were used for striking or knapping, and they bear the corresponding battering and crushing marks. Complete and fractured pebbles account for 61% of the assemblage (n = 17), which means that most of the Lower-TD6.3 material is percussive. This may explain the dominance of fluvial raw materials in this group, particularly quartzite (*c*. 65%). The lack of flakes suggests that this percussive material was used to break bones or undertake another as yet unclassified activity, rather than to knap cores or make tools.

Complete pebbles are small, with an average size of 83.6x58.7x37.7 mm, and an average weight of 342 g (n = 6). The limestone pebbles are a little larger and have more varied morphologies (prism, discoidal, cylindrical, and oval).

**Fig 5:**
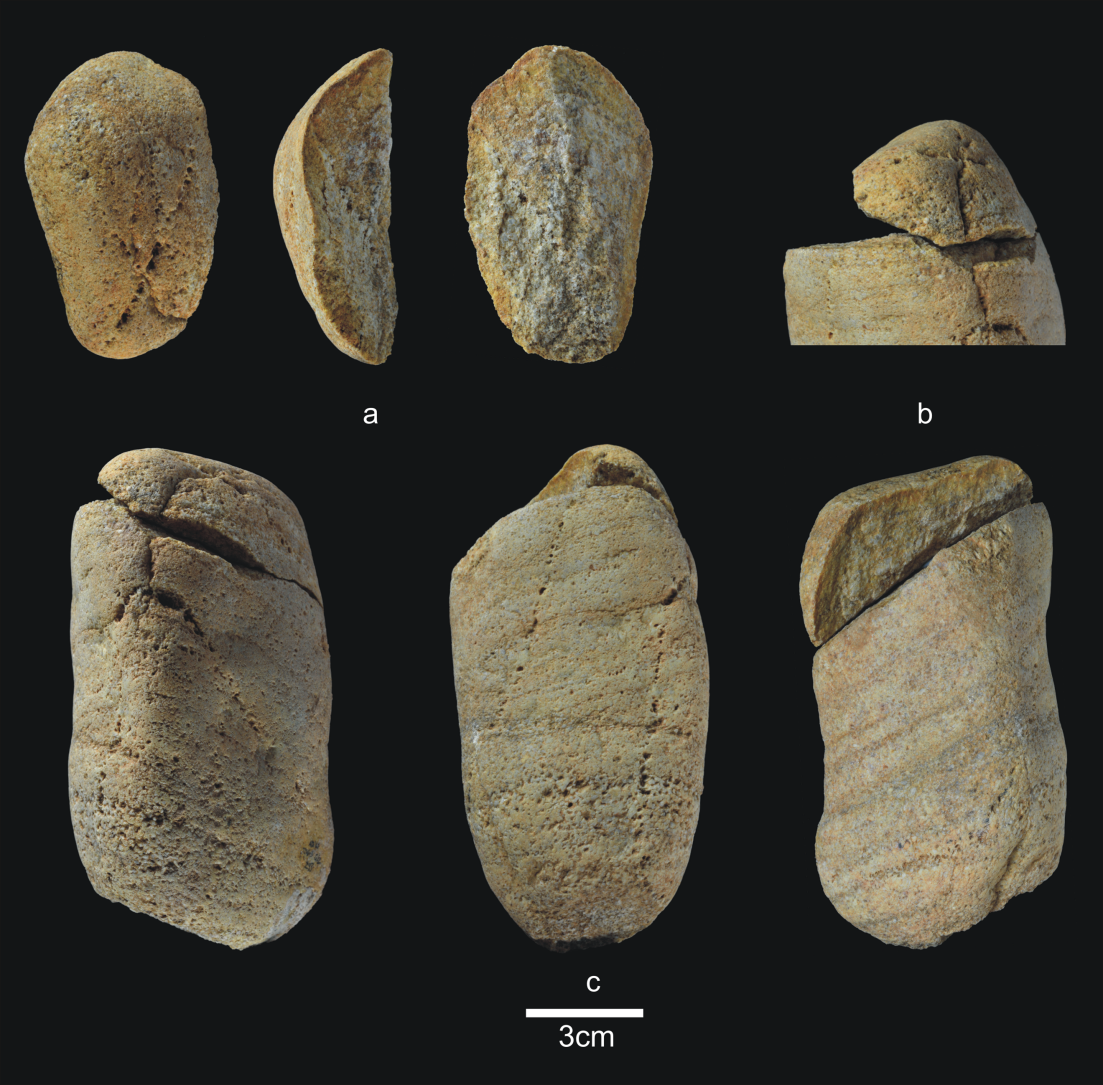
Lithic refits of Lower TD6.3. a) Ata11-F11-97, quartzite pebble fragment; b and c) Refit of Ata11-F11-97 and Ata10-G12-293, quartzite hammerstone.

Lower-TD6.3 contains one refit made up of a broken hammerstone and a fragment of quartzite pebble with percussion marks (micro-conglomerate) (Fig 5) (see the Refits section). This fracture very likely occurred as a result of percussion.

Despite the scarcity of pieces, the variety of raw materials is similar to that found in the lower units at Gran Dolina (i.e., TD4-5), although lacking Cretaceous chert.

In our view, this lithic assemblage represents a marginal hominin occupation, mainly comprising pebbles transported into the cave. Based on their fracture and percussion marks, these pebbles were used but not knapped. The occupation of the cave by other animals, evidenced among other things by the presence of 126 hyena coprolites, is probably responsible for the faunal accumulation, with hominins being merely marginal and sporadic visitors [72].

#### Upper-TD6.3

**Fig 6:**
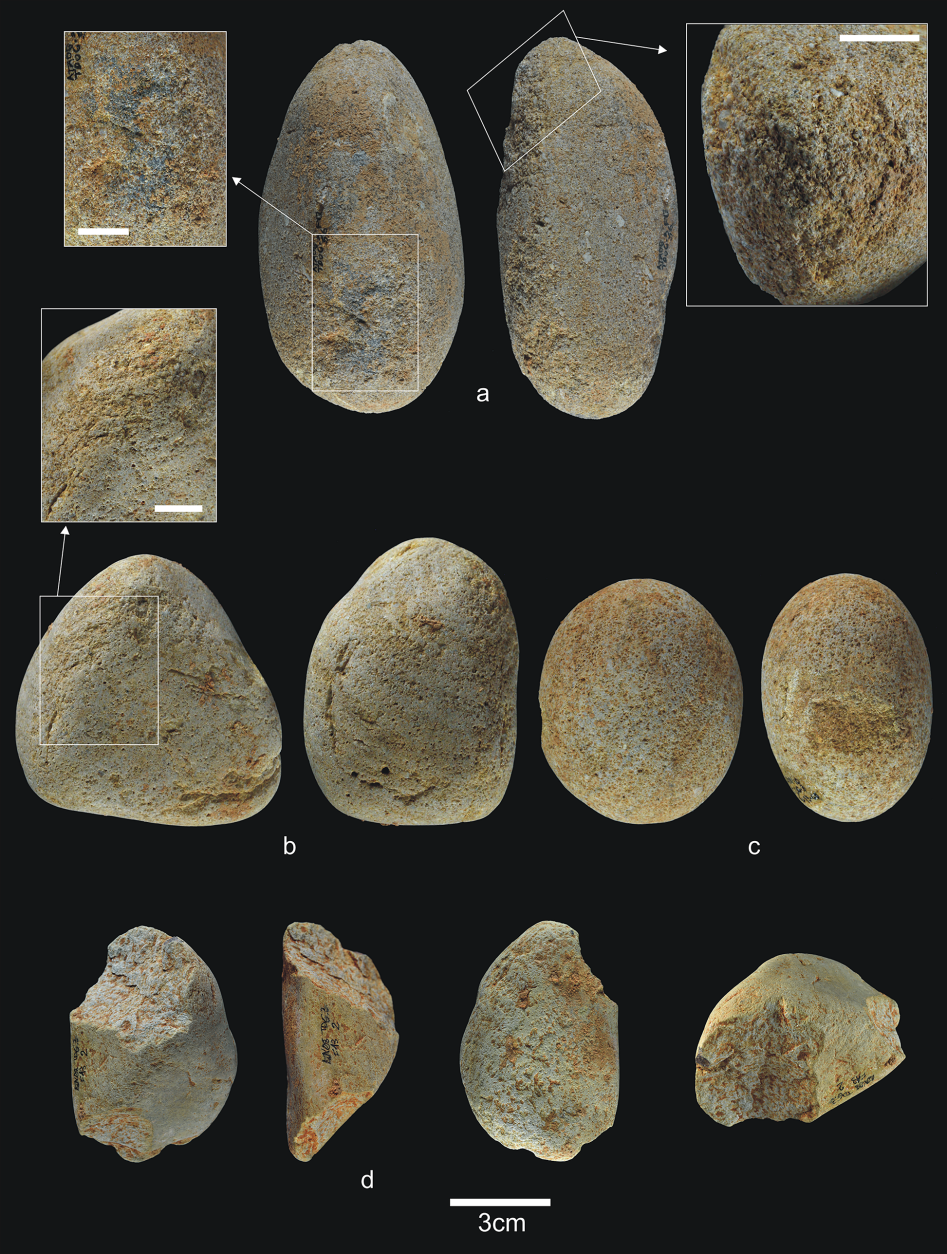
Lithic remains from Upper TD6.3. a) Ata08-F13-56, sandstone hammerstone, and details of percussion marks; b) Ata08-F13-57, quartzite hammerstone; c) Ata10-G13-68, quartzite hammerstone; d) Ata08-F13-2, sandstone core. Scale bar in details = 1cm.

**Fig 7:**
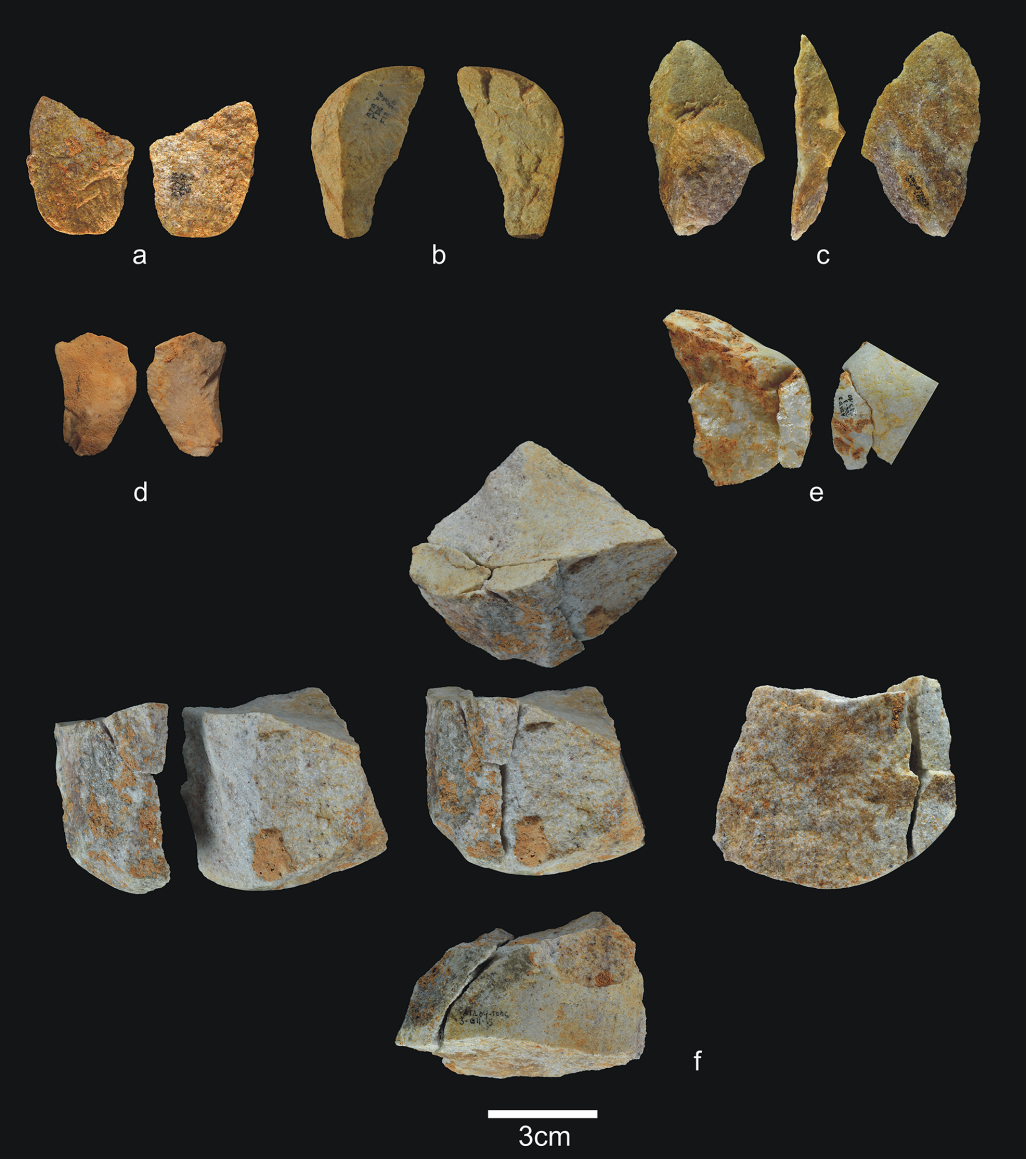
Flakes and refits from Upper TD6.3. a) Ata08-E13-38, sandstone flake; b) Ata08-F12-70, quartzarenite flake; c) Ata08-G14-102, quartzite flake; d) Ata96-H16-423, limestone flake; e) Refit of Ata08-F13-9 (quartz core) and Ata08-F13-10 (quartz flake fragment); f) Refit of Ata09-G11-15 (quartzarenite core) and Ata08-F12-1 (quartzarenite broken flake).

This second stage, the remainder of this subunit, includes 1,305 faunal remains (40 antler fragments, 51 teeth, 1,163 bones, and 51 coprolites) and 56 lithic pieces (Table 3) (Figs 6 and 7). There are no hominin remains.

**Table 3:** Number and type of lithic items.

Upper-TD6.3	Sand	Lime	Qrtz	Qz	Qrtza	Chert	Cret	Neog	Total
**Pebble**	2	2	1		1				**6**
**Hammerstone**	3	1	3		2				**9**
**Fractured pebble**	3	3	1		1				**8**
**Fragment of pebble**					1				**1**
**Core**	1			1	1				**3**
**Core fragment**								1	**1**
**Retouched flake**								1	**1**
**Flake**	2	2	1		3		1	1	**10**
**Fractured flake**			2		3		1		**6**
**Fragment of flake**				1					**1**
**Fragment/chunk**				2				2	**4**
**Indet.**	1					2		3	**6**
**Total**	**12**	**9**	**8**	**8**	**4**	**12**	**2**	**2**	**56**

Indet: Indeterminable pieces due to their poor preservation. Sand: sandstone; Lime: limestone; Qrtz: quartzite; Qz: Quartz; Qrtza: quartzarenite; Ch: indeterminate chert; Cret: Cretaceous chert; Neog: Neogene chert.

Percussive material, including pebbles with no battering marks, still dominates the assemblage (43%), although it is less frequent in Upper-TD6.3 than in Lower-TD6.3, due to the existence of produced flakes and other tools. The complete pebbles are similar in size than those from Lower-TD6.3 (average size 76x56x41 mm; average weight 280 g), although the largest are sandstone, and the smallest are quartzarenite. The morphologies vary, although oval pebbles are particularly common. Most fractured pebbles and fragments of pebbles also bear percussion marks. One has also been slightly flaked as a core. Production processes are reflected by the presence of three cores and one core fragment, along with 18 knapping products.

The three complete cores are of fluvial materials and have undergone unipolar longitudinal knapping, unifacial in two cases and bifacial in one. Two of these were knapped using the bipolar-on-anvil technique. The core fragment is made of Neogene chert and seems to have been flaked using the unifacial unipolar longitudinal strategy.

The only retouched flake is a carinated denticulate from a Neogene chert fragment.

Complete flakes average 38x30x11 mm in size, with no item being smaller than 20mm in any dimension. Some of these may actually be fragments of hammers. The percentage of fractured flakes and flake fragments is the highest (41% of the group of flakes) seen in the entire lithic assemblage from TD6. They mainly comprise diverse quality fluvial materials, which may point to the limited workability of these materials, as well as poor hominin knapping skills. Technically, the flakes may retain some cortex on their dorsal surfaces, have a mean of two dorsal scars and flat butts, which are natural (cortical) or unifaceted, and have great variety of flaking angles (between 85° and 140°), and ventral faces with varied delineations and diffuse bulbs. The morphology of the complete flakes is also varied.

Upper-TD6.3 contains two groups of refits: one core and one flake fragment of quartz from a bipolar-on-anvil knapping episode, and one core and one fractured flake of quartzarenite from a knapping process (Fig 7E and 7F) (see the Refits section).

The presence of hominins at Gran Dolina undoubtedly increased during this stage, given: 1) the greater number of lithic remains found in consecutive layers; 2) the presence of cores and flakes that reflect production; and 3) the diversification of the lithic categories, including the first retouched piece from Gran Dolina.

### TD6.2

The majority of the archaeopalaeontological assemblage of TD6 is concentrated in this subunit. TD6.2 is a complex deposit of channel *facies*, mud layers and floodplain and debris flow *facies* [1, 36]. Although the deposit shows strong lateral discontinuities, the archaeological correlation among the three areas of the site is possible: western, central and eastern (see Table 1).

#### TD6.2.4

**Fig 8:**
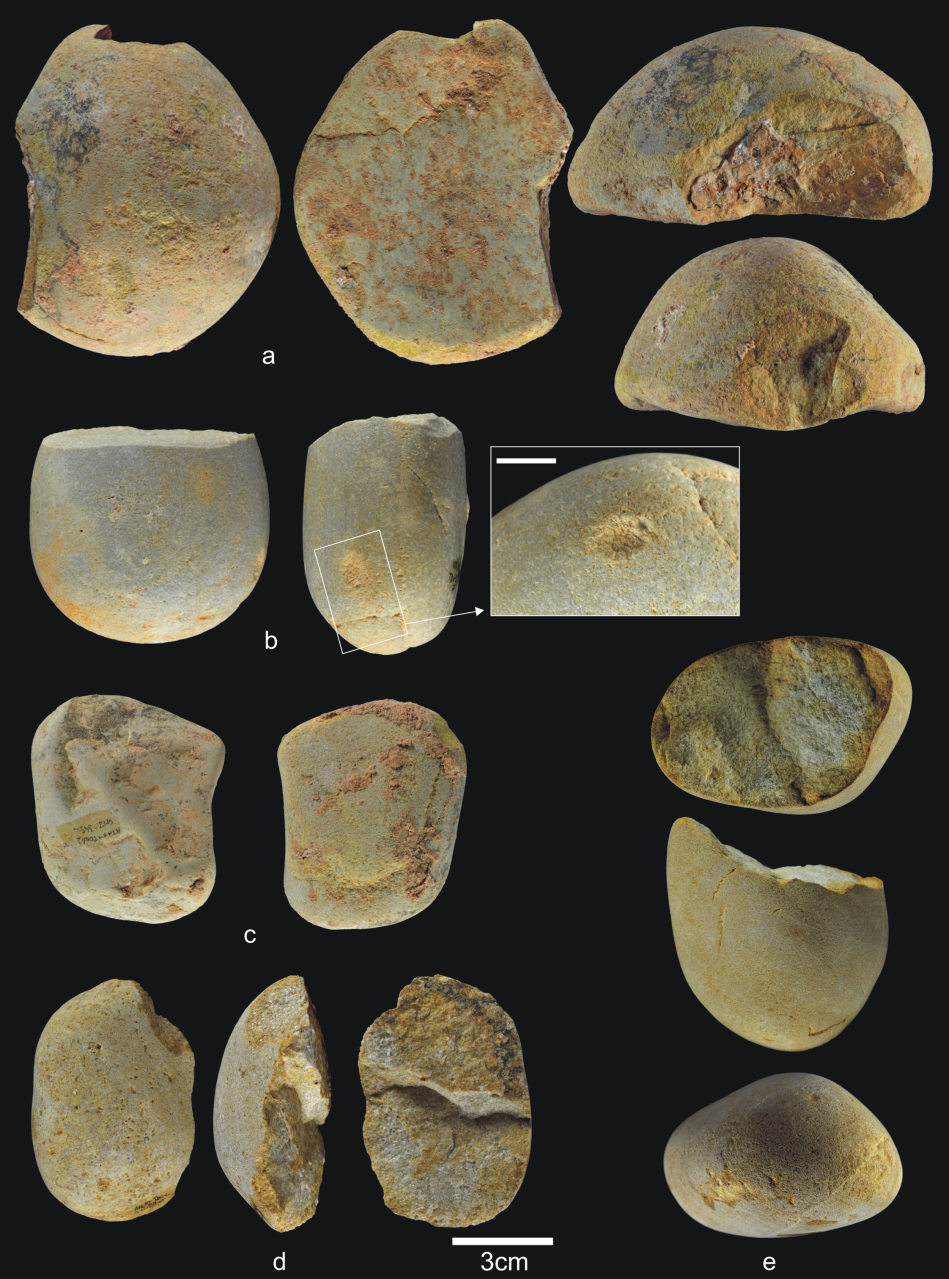
Percussive material from TD6.2.4. a) Ata04-F14-122, broken quartzarenite anvil; b) Ata05-F14-76, quartzite hammerstone and detail; c) Ata09-G12-345, sandstone hammerstone; d) Ata09-G12-410, fragment of quartzarenite hammerstone; e) Ata09-G12-409, fractured sandstone hammerstone. Scale bars in details = 1cm.

**Fig 9:**
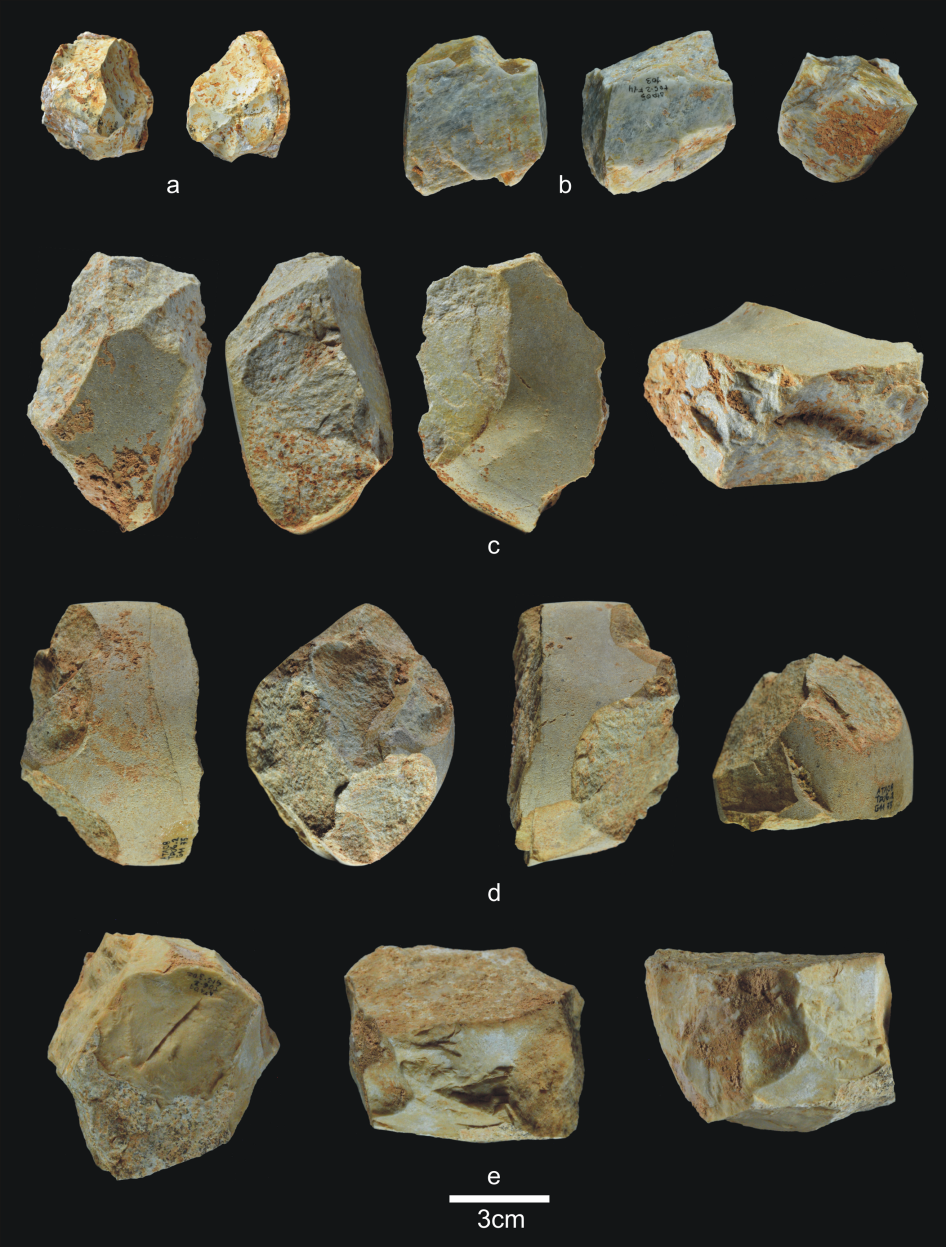
Cores from TD6.2.4. a) Ata04-F14-96, bifacial centripetal, Cretaceous chert; b) Ata05-F14-103, unifacial unipolar longitudinal, quartz; c) Ata08-F12-32, unifacial unipolar longitudinal, quartzite; d) Ata08-G11-75, bifacial unipolar longitudinal, sandstone; e) Ata09-G12-302, unifacial unipolar longitudinal, limestone.

**Fig 10:**
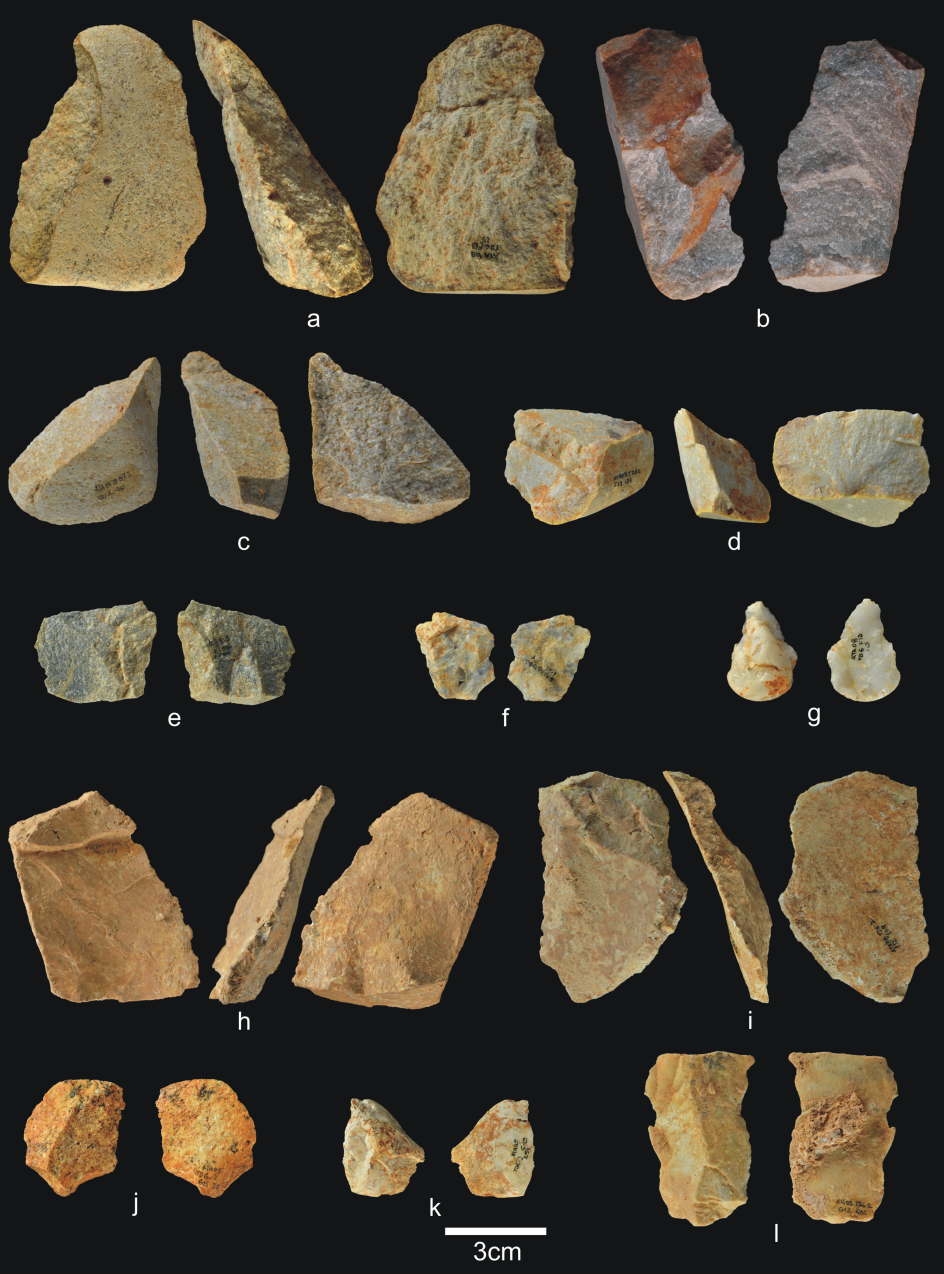
Simple flakes from TD6.2.4. a) Ata08-F13-25, quartzarenite; b) Ata95-I16-166, quartzite; c) Ata09-G12-316, quartzite; d) Ata08-F13-134, quartzite; e) Ata07-F13-559, quartzite; f) Ata08-G11-74, quartz; g) Ata08-F13-45, quartz; h) Ata09-G13-567, Neogene chert; i) Ata08-F12-159, Neogene chert; j) Ata05-G15-211, Neogene chert; k) Ata05-G15-259, Cretaceous chert; l) Ata09-G12-402, limestone.

**Fig 11:**
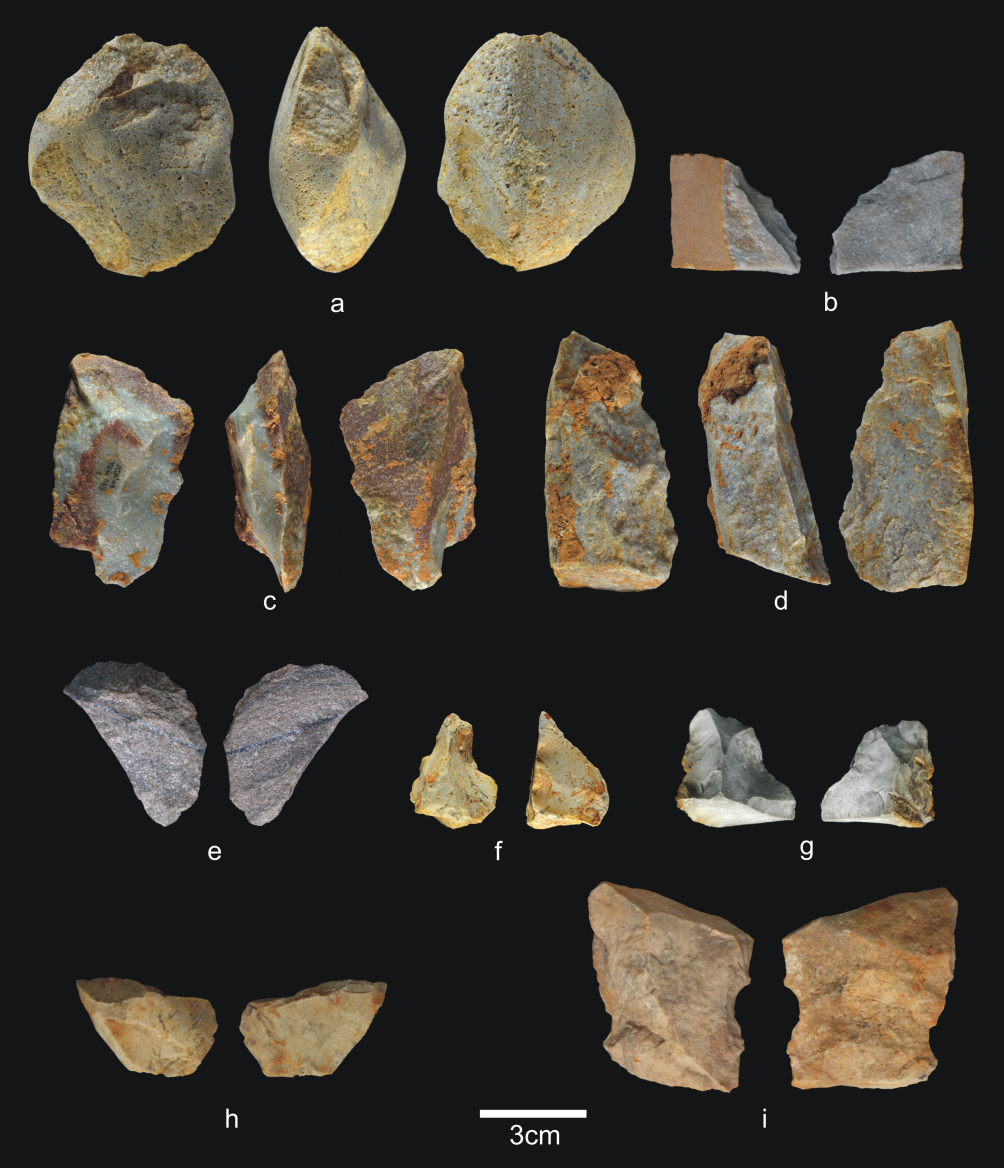
**Shaped tool (a) and retouched flakes from TD6.2.4**. a) Ata05-G15-19, quartzarenite; b) Ata95-G18-83, denticulate sidescraper, quartzite; c) Ata08-E13-1, denticulate sidescraper, quartzite; d) Ata09-G13-528, denticulate sidescraper, quartzite; e) Ata95-G16-163, denticulate sidescraper, quartzite; f) Ata09-G11-10, denticulate point, Neogene chert; g) Ata95-I17-84, denticulate carinated sidescraper, Cretaceous chert; h) Ata96-H16-370, simple point, limestone; i) Ata95-G17-243, possible notch, limestone.

**Fig 12:**
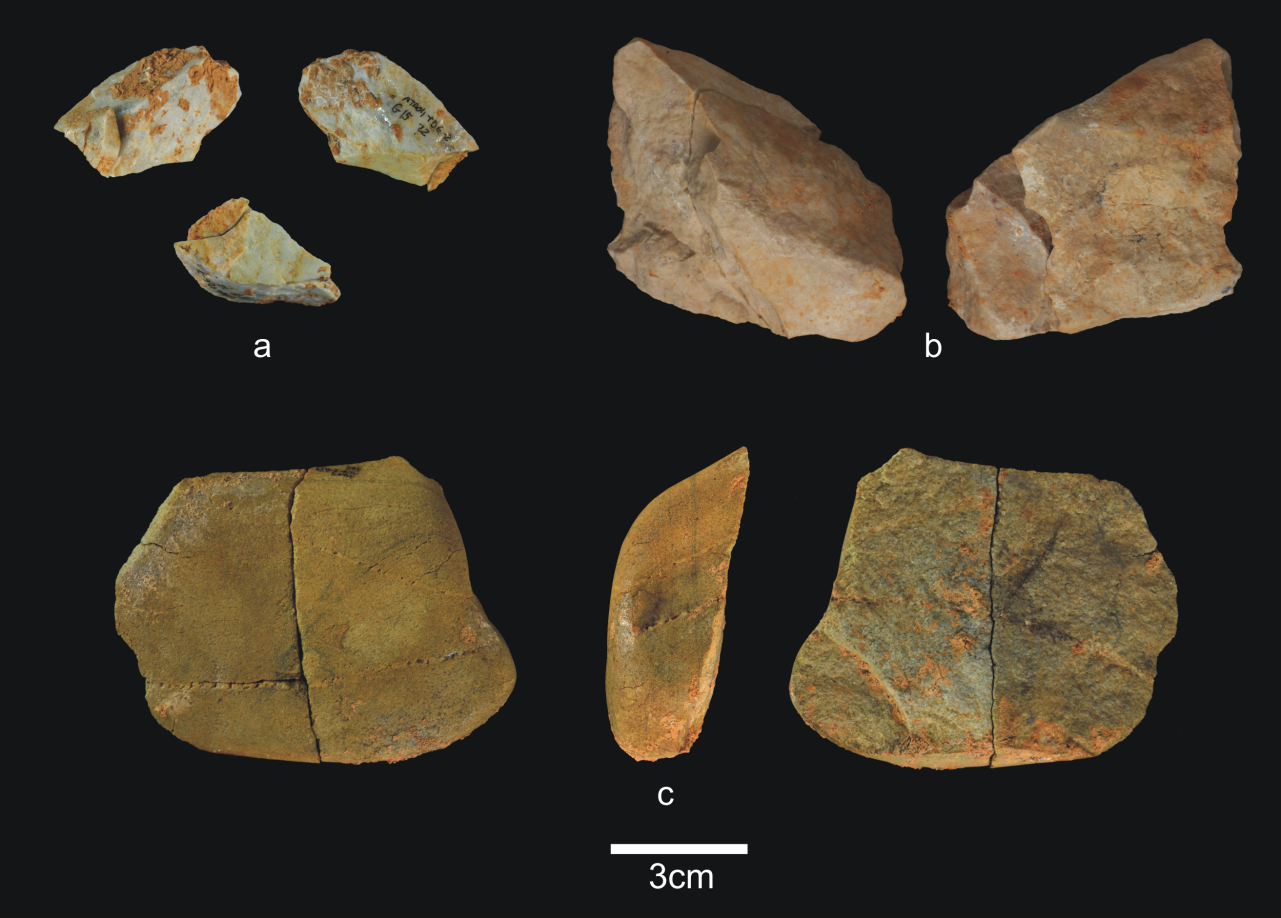
Refits from TD6.2.4. a) Ata04-G15-22 and Ata05-F14-4, quartz; b) Ata95-G17-243 and Ata95-G17-249 limestone; c) Ata09-G13-562 and Ata09-G12-374, quartzarenite.

This is the oldest layer of subunit TD6.2, and is composed of gravitational inputs [1]. It contains 1,712 archaeo-paleontological remains (37 antler fragments, 72 teeth, 1,340 bones, and 15 coprolites), 236 lithic pieces (Table 4) (Figs 8–12) and 12 hominin remains (5 teeth and 7 bones). There are two infant teeth, one of them belonging to Hominin 9. One rib belongs to an adult individual.

**Table 4:** Number and type of lithic items from TD6.2.4.

TD6.2.4	Sand	Lime	Qrtz	Qz	Qrtza	Chert	Cret	Neog	Total
**Pebble**	1	3		1	3				**8**
**Hammerstone**	2	1	9						**12**
**Fractured pebble**	5	3	1		3				**12**
**Fragment of pebble**					2				**2**
**Cores**	2	1	3	1	1		2	1	**11**
**Tool on pebble**					1				**1**
**Retouched flake**		2	5				2	3	**12**
**Flake**	5	18	8	9	9		7	31	**87**
**Fractured flake**	3	2	1	3	6		1	1	**17**
**Fragment of flake**	3		2	1	2			3	**11**
**Fragment/chunk**	2			1				2	**5**
**Indet.**	7	1				2		48	**58**
**Total**	**30**	**31**	**29**	**16**	**27**	**2**	**12**	**89**	**236**

Indet: indeterminable pieces due to their poor preservation. Sand: sandstone; Lime: limestone; Qrtz: quartzite; Qz: Quartz; Qrtza: quartzarenite; Chert: indeterminate chert; Cret: Cretaceous chert; Neog: Neogene chert.

This subunit contains more of all the generic raw material varieties (i.e. microconglomerate, quartzite from the Utrillas formation, orthoquartzite, and metasandstone) [70], indicating a greater range in the search for raw materials, although evidence points to some degree of selection of better quality varieties [63]. Despite this, Neogene chert dominates the lithic assemblage (c. 38%) for the first time in Gran Dolina, a trend that remains constant at this and most other Sierra de Atapuerca Pleistocene sites.

Subunit TD6.2.4 contains notably less percussive material than in other subunits (Fig 8), representing only 14.4% of the whole assemblage. Complete pebbles are smaller than in previous layers (average size 70x53x40 mm; average weight 317 g), although the largest are limestone and quartzite, and the smallest are sandstone and quartzarenite. The morphologies vary, with oval, polyhedral and prismatic items. As in previous layers, most fractured pebbles and fragments of pebbles show percussion marks. One of these hammerstones was flaked as a core, and another complete pebble was used as an anvil. The decrease in percussive material reinforces the more productive character of the hominin occupation at TD6.2.4. This increased production is represented by 11 cores (4.6%), as well as 127 flakes, broken flakes, flake fragments and flake-blanks from retouched pieces, accounting for 53.4% of the assemblage. Compared to the previous marginal occupation of the cave, these figures point to more intense hominin activity. The technological record is also more homogeneous here, and all the stages of the operative chains are represented, meaning that all types of pieces typically produced during knapping are present.

The 11 cores are made of all of the different raw materials (Fig 9), reinforcing the increased impact of the hominin occupation, as well as the diversification of the knowledge and spatial control of available resources. Whatever the raw material, knapping was both unifacial and bifacial, and usually unipolar longitudinal, with four cores showing a centripetal trend and one bipolar-on-anvil in quartz. In the majority of cases, at least 2/3, and sometimes all, of the core surface was used, particularly in the centripetally knapped pieces. However, half were abandoned at an intermediate stage of production. In other words, based on the size of the scars on their surfaces the cores could have produced further flakes. The average size of these cores is 69x58x43 mm, with a standard deviation of 22 cm.

The group of flakes (Fig 10) contains 115 items, including complete flakes, broken flakes and flake fragments. These come from all the raw materials, mainly comprising Neogene chert (31%), followed by limestone, quartzarenite, quartz, quartzite, sandstone, and Cretaceous chert, none of which accounts for more than 16.5% of the whole group. The percentage of fractured flakes is lower than in other subunits at 25%, although this is still high for fluvial materials.

Nineteen complete flakes are smaller than 20 mm, and 10 are larger than 60 mm, but none exceeds 85 mm. The average size is 36x32x14 mm. Technically, there is an even distribution of between zero and two dorsal scars. They are quite variable with regard to the dorsal cortex, which is particularly well preserved in the quartzite items. Butts are flat, unifaceted or natural (cortical), and non-cortical for limestone, sandstone, and Neogene and Cretaceous chert, while they are cortical for the majority of the fluvial items. Bulbs are equally marked and diffuse; the ventral delineations are vary widely, although they are predominantly straight. The flaking angles vary between 85° and 135° for all raw materials. Three Neogene flakes stand out because of their thinness and length, and the fact that their flaking axes have the same direction as their dorsal scars. They belong to the same raw material blank, and are therefore perhaps the work of a single knapper.

There are 13 retouched tools (Fig 11), one of which is typologically a small quartzarenite “pebble tool” (65x56x37 mm) with percussion marks on one of its ends (Fig 11A). The remaining retouched tools are made from a wide variety of raw materials. The flake-blanks are not technically different from the simple flakes, with the exception of their larger average size (42x38x19 mm). They include one possible notch, two *épines*, six denticulates, two points (one denticulate, one simple), and one sidescraper. In many cases, the blanks are thick flakes with a natural back.

This layer contains three refits (see the Refits section) (Fig 12): one quartz refit, formed by two complete flakes extracted consecutively in a flaking process (Fig 12A); one limestone refit, formed by a notch and a flake extracted consecutively in a knapping process (Fig 12B); and one quartzarenite refit, formed by one broken flake and a flake fragment from a Siret fracture (Fig 12C).

#### TD6.2.2/3

**Fig 13:**
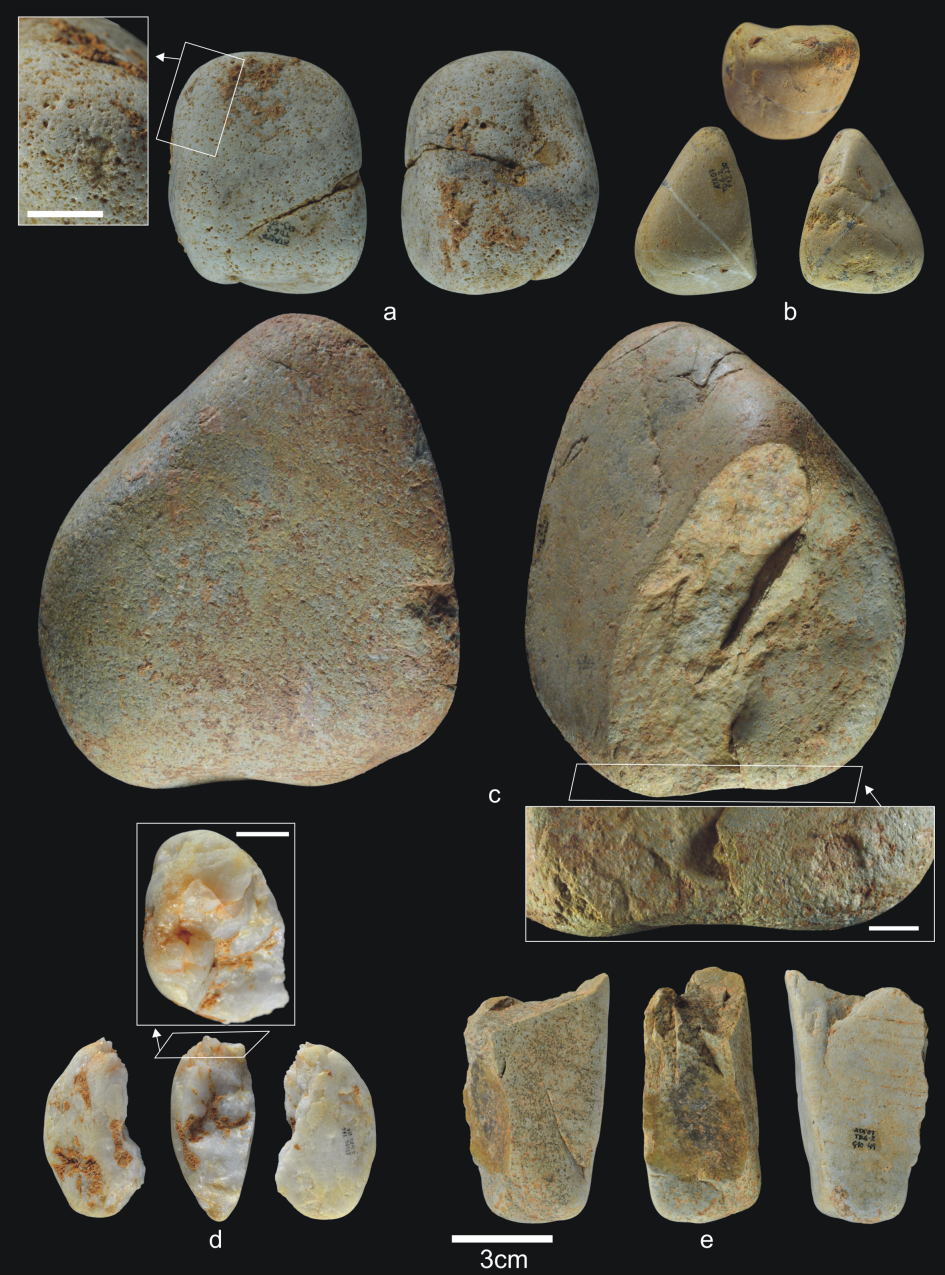
Percussive material from TD6.2.2/3. a) Ata07-E13-60, quartzarenite hammerstone and detail; b) Ata07-F12-230, quartzite hammerstone; c) Ata07-F11-72, fractured sandstone hammerstone and detail; d) Ata09-G03-156, fractured quartz pebble and detail; e) Ata07-G10-49, fractured quartzite hammerstone. Scale bars in details = 1cm.

**Fig 14:**
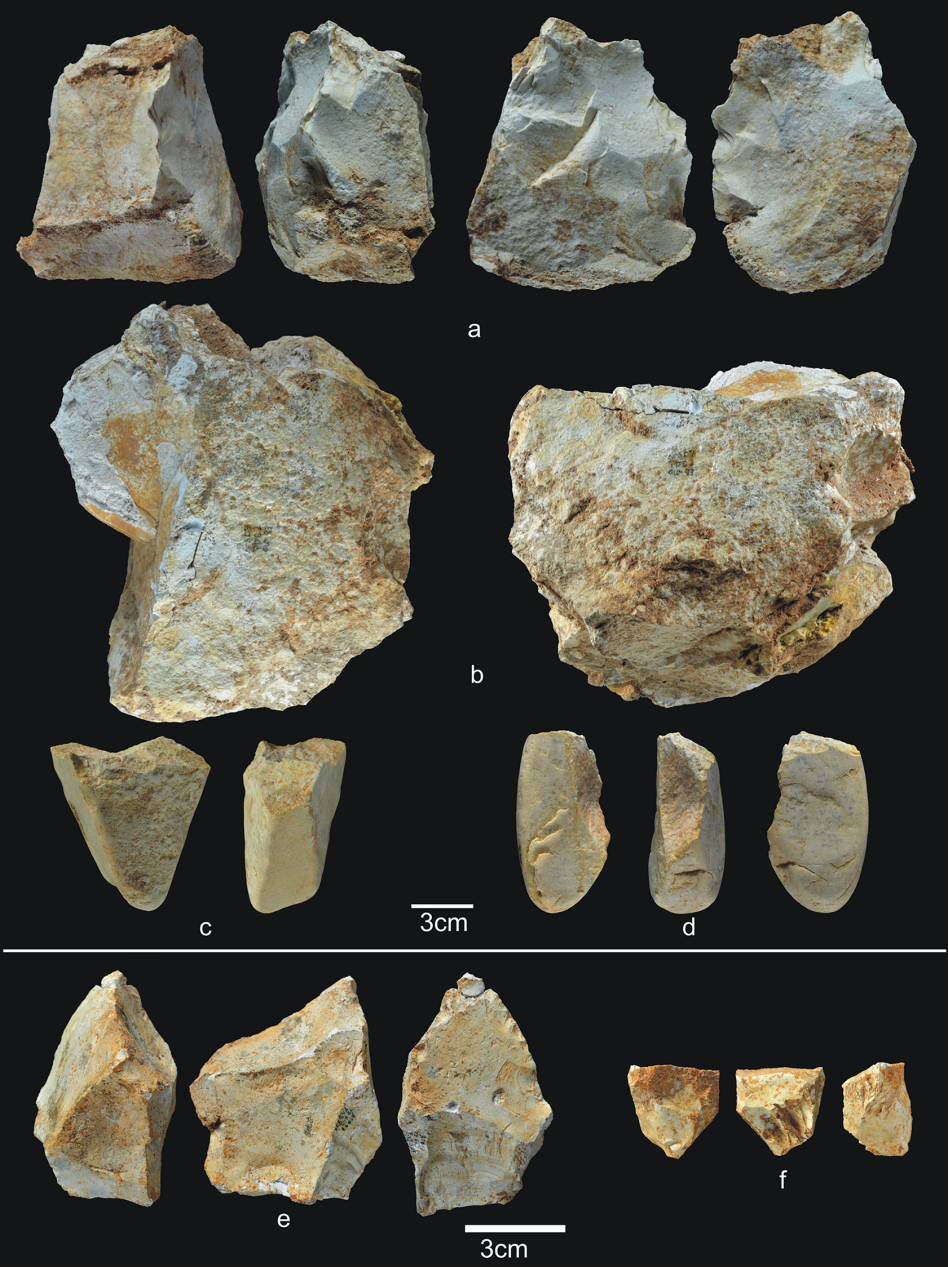
Cores from TD6.2.2/3. a) Ata02-G04-1, multifacial multipolar orthogonal, Neogene chert; b) Ata07-G10-97, bifacial orthogonal, Neogene chert; c) Ata07-G11-122, unifacial bipolar orthogonal, quartzarenite; d) Ata07-G10-73, bifacial unipolar longitudinal, quartzarenite; e) Ata07-F13-526, multifacial multipolar orthogonal, Cretaceous chert; f) Ata09-G04-72, multifacial, Cretaceous chert.

**Fig 15:**
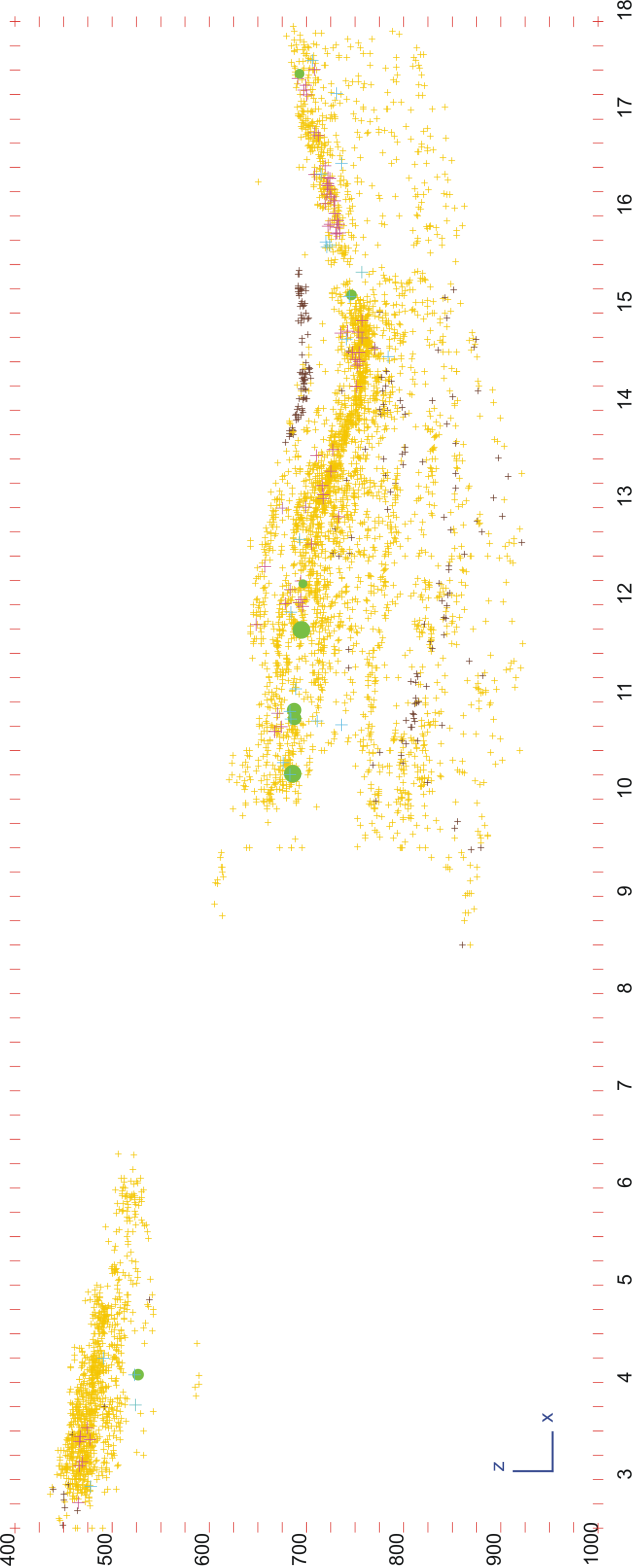
Vertical distribution of archaeo-paleontological remains in TD6. Faunal, hominin and lithic remains of Row G, together with all the large-sized Neogene chert cores of TD6.

**Fig 16:**
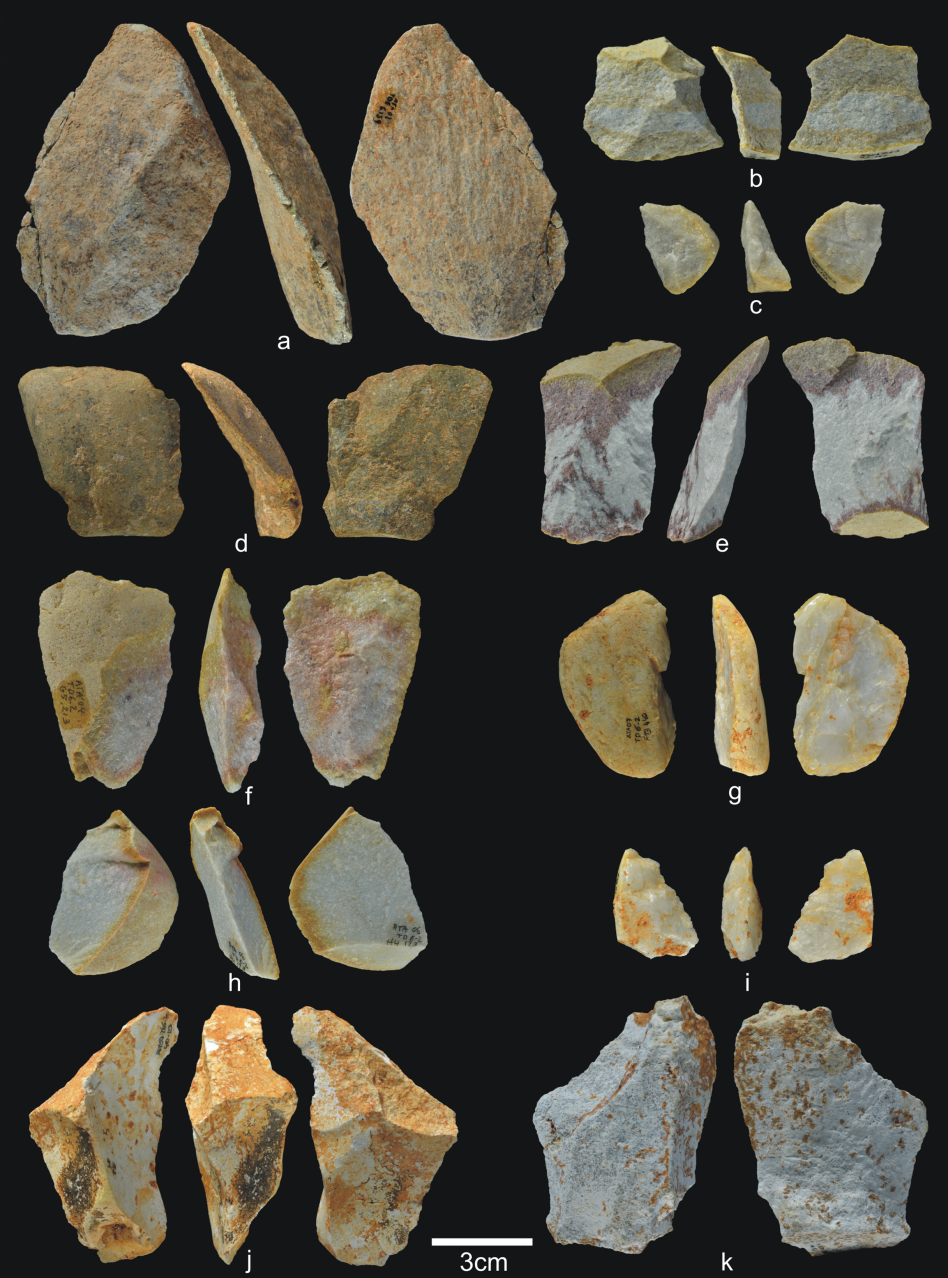
Simple flakes from TD6.2.2/3. a) Ata03-G15-9, sandstone; b) Ata04-G05-169, quartzarenite; c) Ata04-G05-173, quartzite; d) Ata04-G06-49, sandstone; e) Ata04-G05-46, quartzite; f) Ata04-G05-213, quartzite; g) Ata07-F13-440, quartz; h) Ata06-H04-123, quartzite; i) Ata07-F11-79, quartz; j) Ata07-G10-123, Cretaceous chert; k) Ata09-G13-86, Neogene chert.

**Fig 17:**
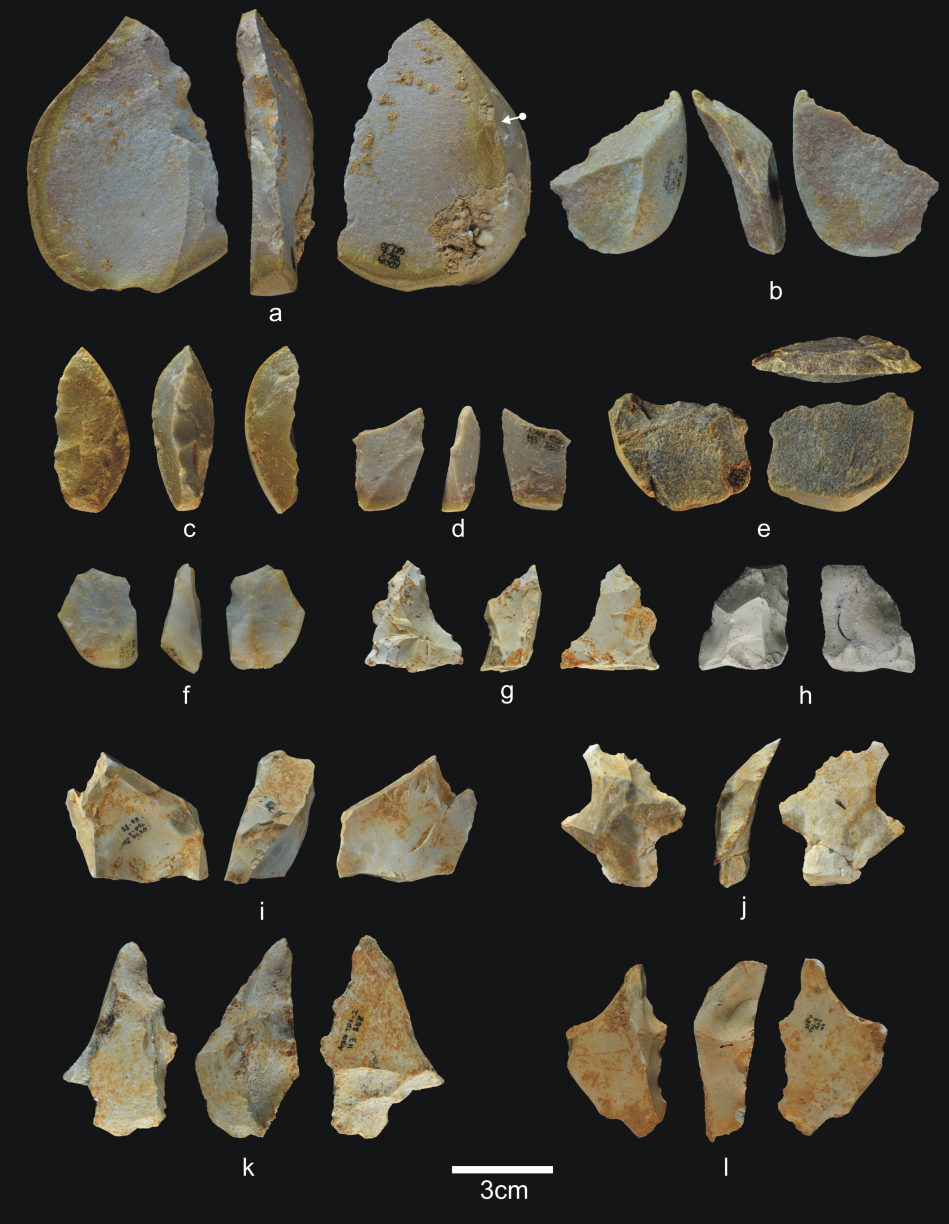
Retouched flakes from TD6.2.2/3. a) Ata09-G12-20, denticulate sidescraper, quartzite; b) Ata04-G06-13, *épine*, quartzarenite; c) Ata09-G04-50, denticulate carinated sidescraper, quartzite; d) Ata06-H03-252, abrupt-retouched flake, quartzite; e) Ata09-G03-247, denticulate sidescraper, quartzite; f) Ata06-H03-174, notch, quartz; g) Ata03-G15-59, notch, Neogene chert; h) Ata95-G18-50, lateral-transversal sidescraper, Cretaceous chert; i) Ata06-H03-88, abrupt-retouched flake, Cretaceous chert; j) Ata11-G04-1, denticulate sidescraper, Cretaceous chert; k) Ata06-H03-258, denticulate carinated sidescraper Neogene chert; l) Ata11-G03-44, denticulate carinated point, Cretaceous chert.

**Fig 18:**
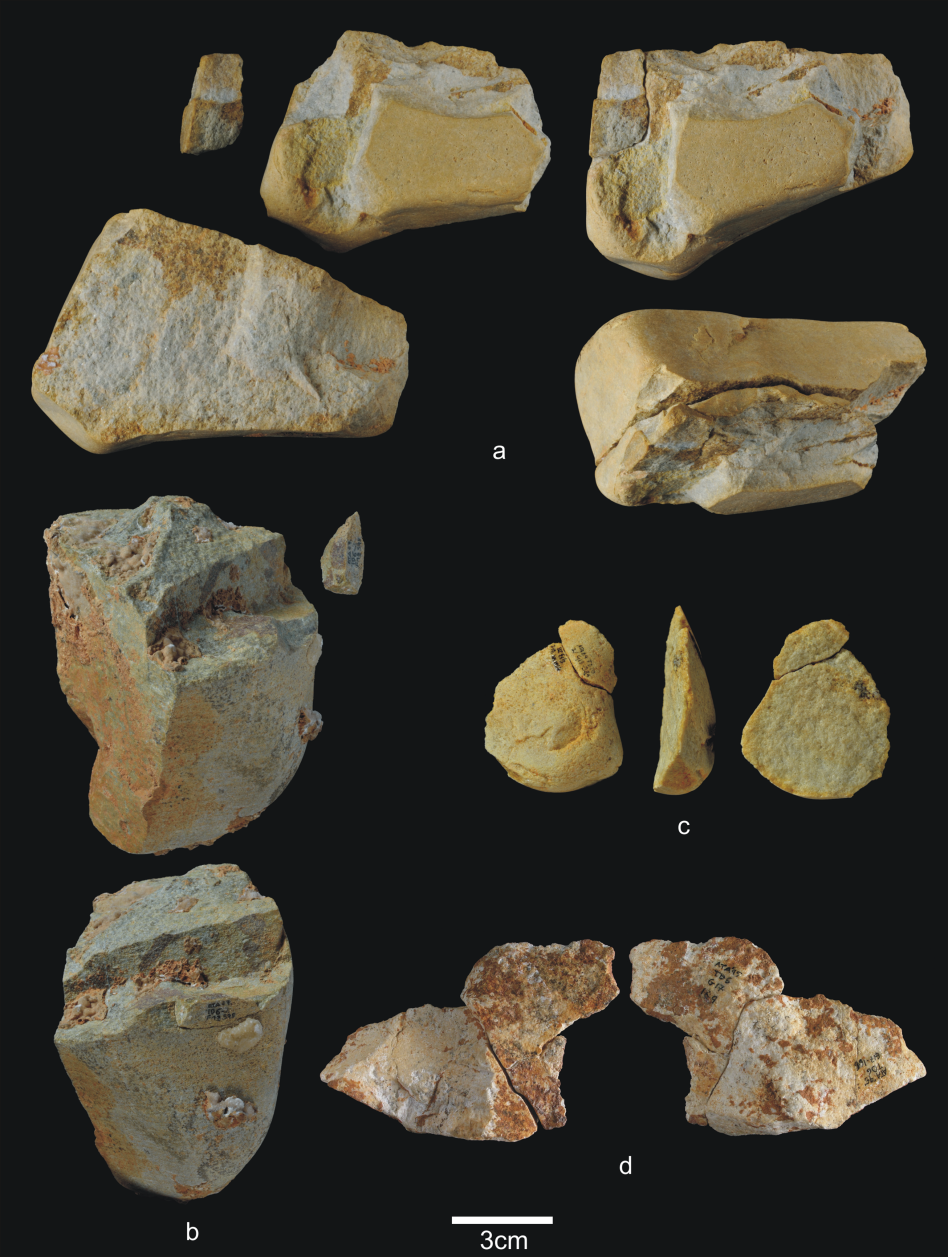
Refits from TD6.2.2/3. a) Ata95-H17-137 + Ata03-G11-1 + Ata07-F12-147, sandstone; b) Ata07-E13-130 and Ata07-F13-395, orthoquartzite; c) Ata09-G13-383 and Ata09-G13-384, quartzarenite; d) Ata95-G17-166 and Ata95-G17-169, Neogene chert.

This is the richest deposit of unit TD6, formed by channel *facies* and floodplain and debris flow *facies* [1]. It contains 4,026 archaeo-palaeontological remains: 3,539 faunal remains (127 antler fragments, 158 teeth, 3,240 bones, and 14 coprolites), 388 lithic pieces (Table 5) (Figs 13–18), and 88 hominin remains (5 teeth and 83 bones). Eighteen of the hominin remains belong to adult individuals, 11 to young adult individuals, 15 to young individuals, and 3 to infants. In addition, Hominin 3 (*c*. 11 years old), Hominin 4 (juvenile) and Hominin 6 (juvenile) belong to this layer. None of the hominin refits found to date are more than 15 cm away one from each other.

**Table 5:** Number and type of lithic items from TD6.2.2/3.

TD6.2.2/3	Sand	Lime	Qrtz	Qz	Qrtza	Chert	Cret	Neog	Total
**Pebble**	1	1		1					**3**
**Hammerstone**		3	2		3				**8**
**Fractured pebble**	4	2	2	1	1				**10**
**Fragment of pebble**	2		1		4				**7**
**Core**	1	1	2	1	3		2	5	**15**
**Core fragment.**								2	**2**
**Retouched flake**			6	2	7		13	7	**35**
**Flake**	10	11	17	13	13		29	51	**144**
**Fractured flake**	2	1	8	8	6		3	4	**32**
**Fragment of flake**	2		6	3	2		4	6	**23**
**Fragments/chunks**	7	6	2	6	3		1	6	**31**
**Indet.**	13	1	1	1		3	4	55	**78**
**Total**	**42**	**26**	**47**	**36**	**42**	**3**	**57**	**135**	**388**

Indet: indeterminable pieces due to their poor preservation. Sand: sandstone; Lime: limestone; Qrtz: quartzite; Qz: Quartz; Qrtza: quartzarenite; Ch: indeterminate chert; Cret: Cretaceous chert; Neog: Neogene chert.

The raw materials and varieties are as diverse as in the previous layer, with a dominant use of Neogene chert (c. 35%).

Percussive material (Fig 13) decreases noticeably in this deposit, down to 6.9% of the lithic assemblage and, as in other layers, most fractured pebbles and pebble fragments bear percussion marks. The average size of complete pebbles is similar to that in previous layers (79x63x41 mm), but the average size of the quartzite and quartzarenite items is particularly low (68x41x37 mm and 57x47x32 mm, respectively). One fractured pebble was also used as an anvil. Cores (4.3%) and knapping products (including flake-blanks of retouched flakes) number 234 items (60.3%). Therefore, TD6.2.2/3 continues to display evidence of intense hominin impact, first established in the preceding layer, in which faunal and hominin representation increases, along with the size of the industrial assemblage to process them.

As in the previous layer, the 17 cores (Fig 14) represent all the raw materials found at the site, although the case of one limestone core is questionable. Additionally, the quartz core bears only a single scar. The remainder are dominated by unifacial cores with unipolar longitudinal knapping, particularly of the fluvial materials, but also multifacial cores of Neogene and Cretaceous chert with orthogonal knapping. However, the knapping is not systematic, meaning that most of the cores were only flaked on approximately 25% or less of their productive surface; in other words, they were abandoned during the initial production stages.

The average size of these cores is larger than in previous layers, up to 92x74x60 mm, although this is mainly due to the presence of seven large cores, four of them Neogene chert. Among the latter, the smallest core measures 117x109x85 mm, and the largest is 178x154x131 mm. This is one of the major characteristics of this archaeo-paleontological layer: the presence of huge Neogene chert blanks transported from the surrounding hills to the cave for use as cores, and which are abandoned at an initial or intermediate stage of knapping, after a few successful strikes. There are only two other large cores of Neogene chert in TD6, and these were found in the overlying layer (TD6.2.0–1), as described below. These large cores are distributed throughout the excavated areas of this layer (western, central and eastern) (Fig 15).

Only eight Neogene chert flakes are larger than 60 mm, but there are five Neogene chert cores, four of them measuring more than 115x100x80 mm, all with large and medium-sized scars. This means that an indeterminate number of medium and large flakes extracted from these cores are missing, because they are either still buried in the unexcavated areas of TD6, or they were transported away from the site.

The group of flakes (Fig 16) consists of 199 items (51.3%), including whole and broken flakes, and flake fragments. They are made from all the raw materials, although mainly Neogene chert (30%), followed by Cretaceous chert (18.5%). The percentage of fractures is similar to that in the previous layer, around 29%, and fluvial materials are still the most prone to fracturing. The average size of the whole pebbles (77x60x40 mm) and their average weight (355 g) is no different to that recorded in other layers.

Twenty-four flakes are smaller than 20 mm and 13 are larger than 60 mm, but only one exceeds 100 mm (99x148x36 mm). This large flake was neither retouched nor shaped. The average size of the complete flakes is 34x32x12 mm, similar to the previous layer. Technically, these flakes have two to four dorsal scars; most are non-cortical on their dorsal surfaces, regardless of the raw material. Butts are mainly flat, unifaceted or natural (cortical), and ventral delineations are varied. Flaking angles range between 65° and 135° in all the raw materials. Trapezium morphologies dominate although there is a wide variety of shapes.

Meanwhile, the production of retouched flakes (Fig 17) increases in this layer (8.6%), a feature that is constant upwards through the stratigraphic sequence of TD6. There are 35 pieces, made from all the raw materials, but Cretaceous chert dominates (37%), followed by Neogene chert (20%) and quartzite (20%). Interestingly, in TD6.2.2/3 there are 57 pieces of Cretaceous chert (cores, flakes, etc.), 13 of which are retouched flakes (23%). As in the rest of the layers of TD6, retouched flakes were often produced using Cretaceous chert, pointing to the differential use of these materials in the production of shaped tools.

The density of retouched flakes in the western area of the occupation is actually high (18 items/3m^2^ approx.), compared to the central (8 items/c.9m^2^) and eastern areas (Test Pit) (21 items/c.7m^2^). The western and central areas also contain most of the faunal remains, with twice as many as were found in the Test Pit. However, the western area yielded fewer hominin remains.

The 35 retouched flakes are similar in average size to the simple flakes (37x37x16 mm) and, technically, do not differ from one other. Retouching is limited to less than 25% of the perimeter, and is simple or semi-abrupt, and straight, convex or concave in delineation. There are eight items with two generations of retouch: The dorsal surface of the piece was modified first and then, retouching was superimposed to delineate the edge. These items were made of Cretaceous chert, Neogene chert, and quartzite. The 35 retouched flakes include two abrupt-retouched pieces, seven side and distal scrapers, one carinated point and 25 denticulates, including five notches, four *épines*, 15 denticulate side-scrapers, and one denticulate point.

There are four groups of refits in TD6.2.2/3 (see the Refits section) (Fig 18), as well as a couple of retouched flakes that seem to belong to the same pebble. The first refit is the most interesting, because it involves three pieces (Fig 18A) that connect the recently excavated central area to the Test Pit, excavated during the 1990s. This refit is a knapping sequence of sandstone comprising one core and two flakes. This knapping sequence was as follows: a series of small flakes were detached from the core; one of these small flakes was found in the Test Pit excavation; a medium-sized flake to which the small flake refits was recovered about 7 m away. Finally, and found 1 m from this medium-sized flake was the core to which it was successfully refit (see Refits section). The second refit an orthoquartzite artefact, and is formed by a core and a fragment of flake from a knapping process (Fig 18B). A third refit is formed by one broken flake and a flake fragment of quartzarenite (Fig 18C). The fourth refit is Neogene chert and comprises two flakes broken diagonally (Fig 18D).

#### TD6.2.0–1

**Fig 19:**
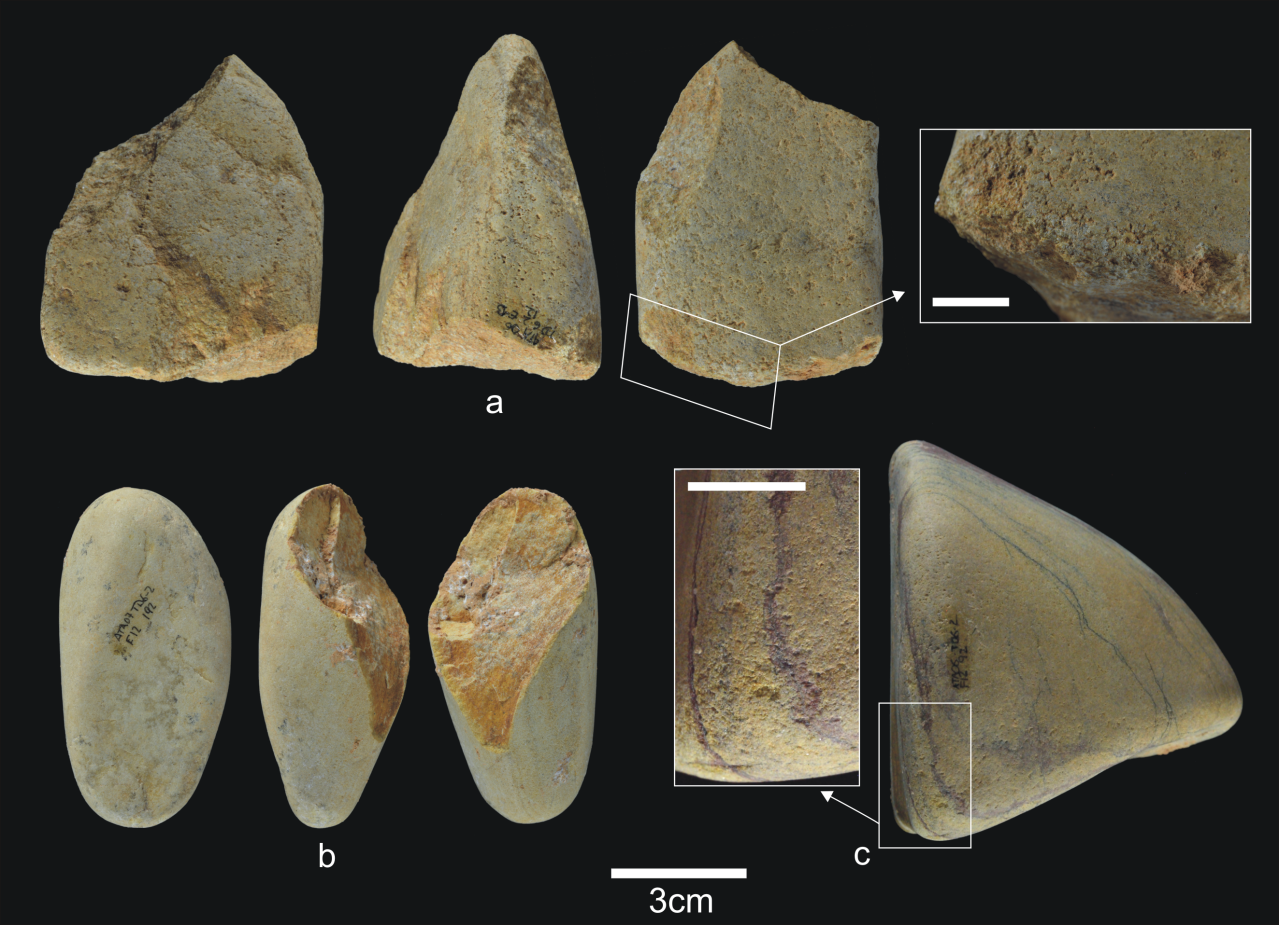
Percussive material from TD6.2.0–1. a) Ata06-E13-12, fractured quartzarenite hammerstone and detail; b) Ata06-F12-92, quartzite hammerstone; c) Ata07-F12-192, fractured quartzarenite pebble and detail. Scale bars in details = 1cm.

**Fig 20:**
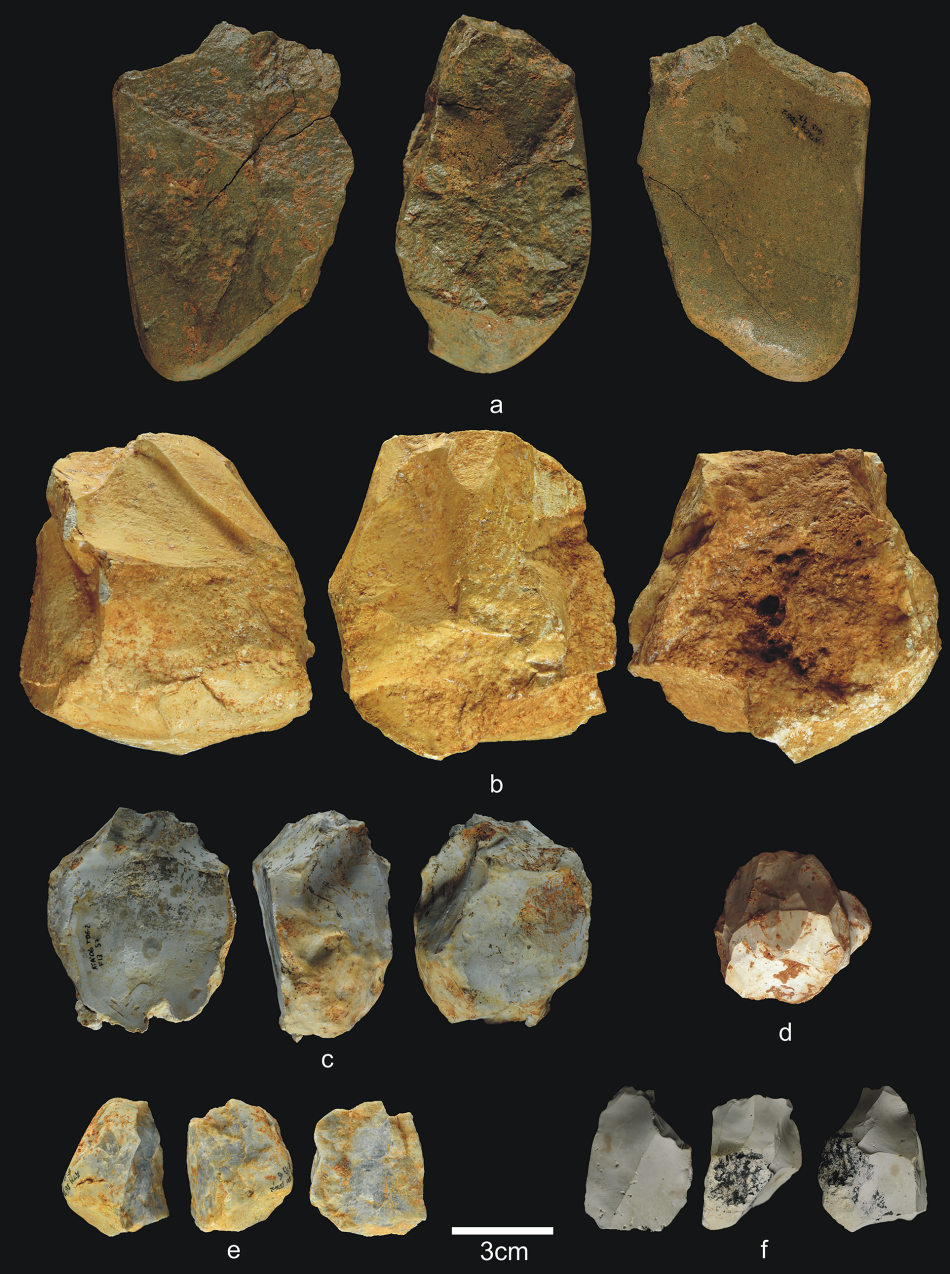
Cores from TD6.2.0–1. a) Ata06-G13-47, bifacial unipolar longitudinal, sandstone; b) Ata94-J17-10, multifacial multipolar orthogonal, Neogene chert; c) Ata06-F13-57, unifacial, two removals, Cretaceous chert; d) Ata94-I18-27, unifacial multipolar centripetal, Cretaceous chert; e) Ata06-E13-6, unifacial unipolar longitudinal, quartz; f) Ata94-I16-38, bifacial multipolar centripetal, Cretaceous chert.

**Fig 21:**
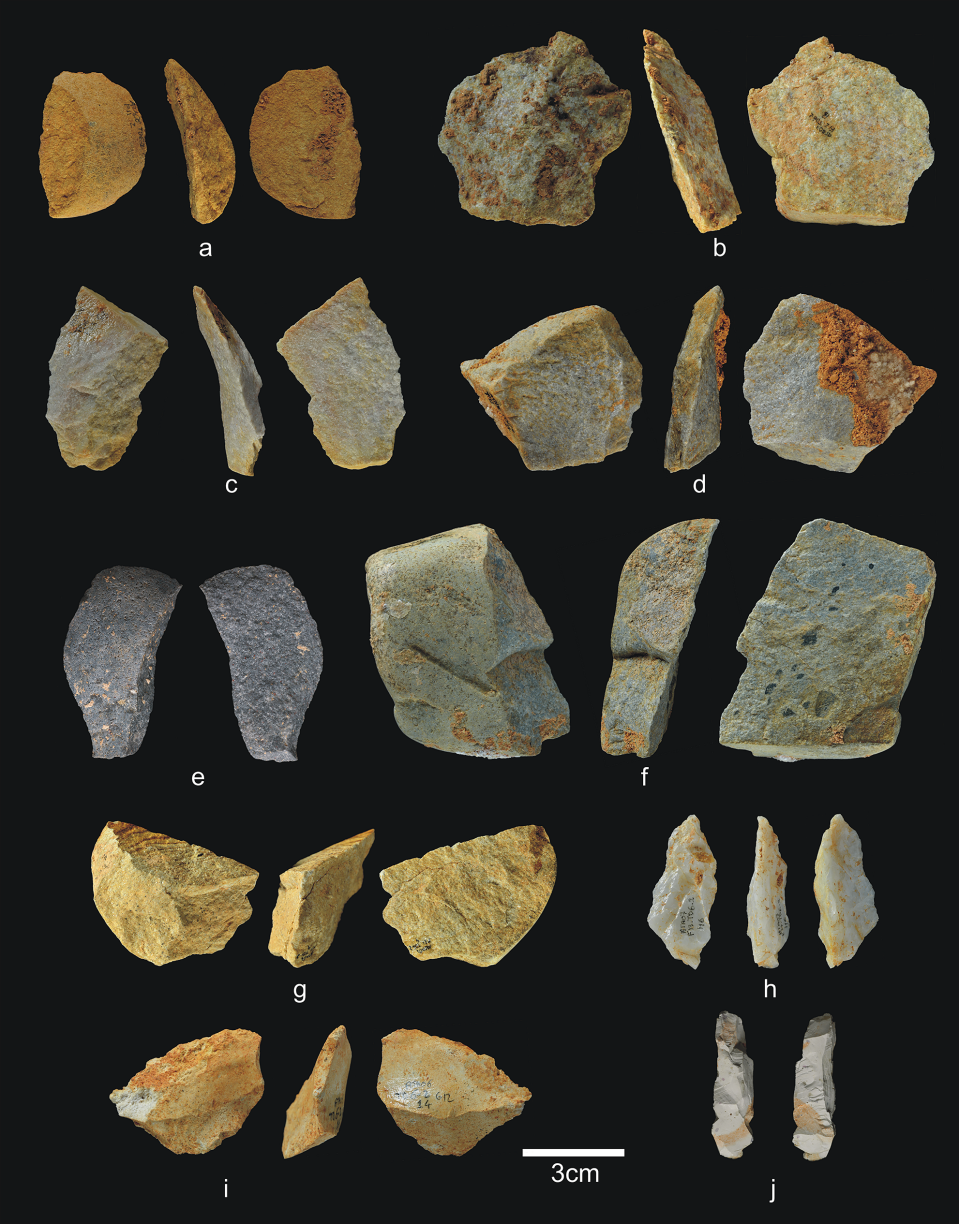
Simple flakes from TD6.2.0–1. a) Ata06-F11-2, quartzarenite; b) Ata07-F12-13, quartzarenite; c) Ata06-F13-58, quartzite; d) Ata06-F12-27, quartzite; e) Ata94-I17-2, quartzite; f) Ata07-G11-112, quartzite; g) Ata07-F11-4, sandstone; h) Ata07-F13-116, quartz; i) Ata06-G12-14, Neogene chert; j) Ata94-I17-31, Cretaceous chert.

**Fig 22:**
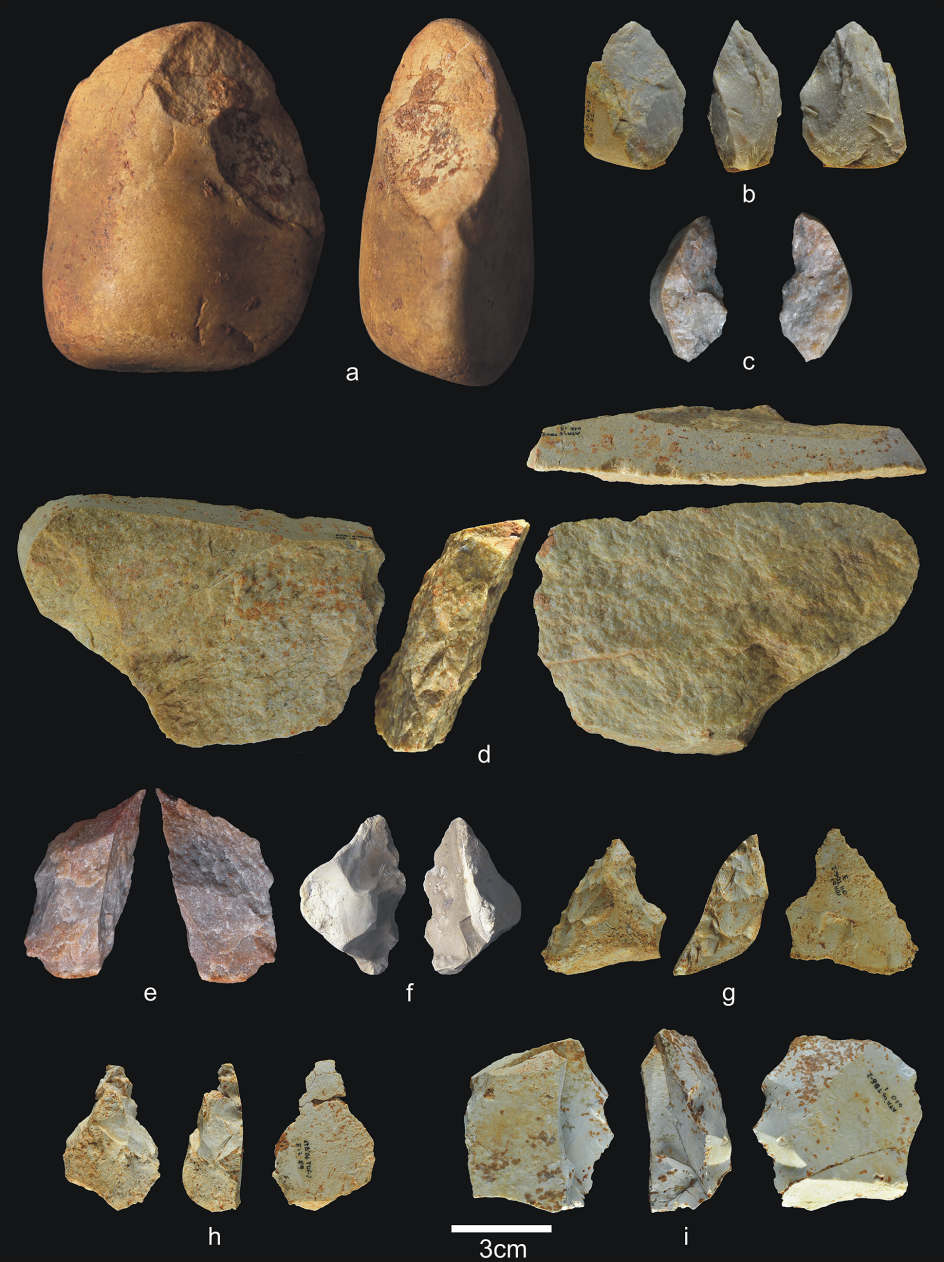
**Chopper (a) and retouched flakes from TD6.2.0–1**. a) Ata94-G17-2, sandstone chopper; b) Ata07-G11-22, denticulate sidescraper, quartzite; c) Ata94-I18-18, marginal denticulate, quartz; d) Ata06-G12-15, denticulate sidescraper, quartzarenite; e) Ata94-J18-51, marginal sidescraper, quartzite; f) Ata94-G17-12, denticulate carinated sidescraper, Cretaceous chert; g) Ata07-G11-3, carinated sidescraper, Neogene chert; h) Ata06-F12-89, Tayac point, Neogene chert; i) Ata06-G10-1, notch, Neogene chert.

**Fig 23:**
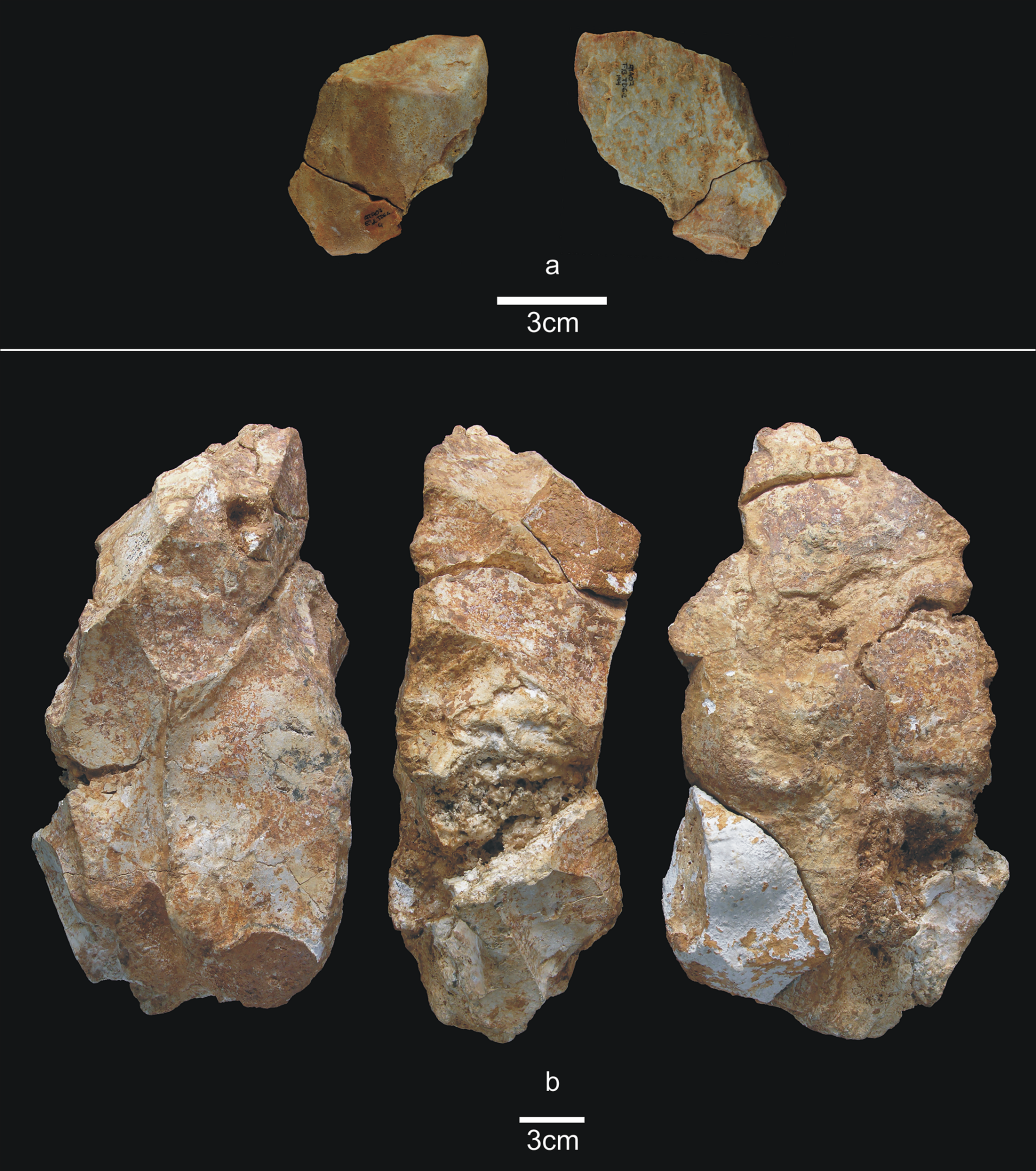
Refits from TD6.2.0–1. a) Ata07-E12-4 and Ata07-F13-144, quartzarenite; b) Ata86-S/C-1 + Ata94-G17-20 + Ata94-G18-1, Neogene chert.

This set of two layers represents the youngest TD6.2 subunit, and it is formed by channel, debris flow and floodplain *facies* [1]. TD6.2.0–1 contains 1,181 faunal remains (26 antler fragments, 7 coprolites, 45 teeth, and 1,103 bones), 64 hominin remains (15 teeth and 49 bones), and 207 lithic items (Table 6) (Figs 19–23). As in the preceding layer, hominin remains belong to individuals of all ages, including four from young individuals and 18 from adults. In addition, Hominin 1 (*c*. 14 years old), Hominin 2 (*c*. 4 years old), Hominin 5 (*c*. 6 years old), Hominin 7 (*c*. 17 years old), and Hominin 10 (juvenile) pertain to this layer. The remains of Hominin 1 (n = 15) were found less than 50 cm away from one another, horizontally, while the two pieces of Hominin 5 were found about 2 m from one another.

**Table 6:** Number and type of lithic items from TD6.2.0–1.

TD6.2.0–1	Sand	Lime	Qrtz	Qz	Qryza	Cret	Neog	Total
**Pebble**		1						**1**
**Hammerstone**			2					**2**
**Fractured pebble**	2	1	3		3			**9**
**Fragment of pebble**			1		1			**2**
**Core**	1	1	1	2	2	4	8	**19**
**Tools on pebble?**	1							**1**
**Retouched flake**	1	1	2	1	3	7	5	**20**
**Flake**	6	4	12	4	9	19	31	**85**
**Fractured flake**	3	1	4	2	6			**16**
**Fragment of flake**	1		1	1	1		2	**6**
**Fragment/chunk**	2	7		3			2	**14**
**Indet.**	1	1				2	28	**32**
**Total**	**18**	**17**	**26**	**13**	**25**	**32**	**76**	**207**

Indet: Indeterminable pieces due to their poor preservation. Sand: sandstone; Lime: limestone; Qrtz: quartzite; Qz: Quartz; Qrtza: quartzarenite; Cret: Cretaceous chert; Neog: Neogene chert.

The raw materials and varieties present are as diverse as in the preceding layers, with the use of Neogene chert once again being predominant (c. 36%).

The percussive material in TD6.2.0–1 (Fig 19) decreases again, although slightly, to 14 items (6.7%), and here not only complete pebbles but also most fractured pebbles and pebble fragments bear percussion marks. One has also been slightly flaked as a core. Cores (9.1%) and their respective products (including all types of flakes and the flake-blanks for retouched pieces) are more prevalent (61%). It seems, therefore, that lithic production intensified at this time, despite the decrease in the absolute number of items in this layer. The average size of the complete pebbles is 84x73x50 mm.

Cores of all raw materials are present (Fig 20), although Neogene chert predominates (42%). There are cores with few scars and the knapping documented here is poorly organised, but this level contains two of the best cases of centripetal cores in the entire TD6 assemblage, both Cretaceous chert (Fig 20D and 20F). Unipolar longitudinal knapping is represented in six cores, mainly Neogene chert. Two quartz pebbles were flaked by bipolar-on-anvil technique. They were abandoned at the final knapping stage. Cores of the other fluvial materials were discarded at an intermediate production stage, as were the Cretaceous chert cores. Finally, Neogene chert cores were abandoned at all stages of production.

As in TD6.2.2/3, TD6.2.0–1 also contains two large Neogene chert cores, scarcely flaked, from large fragments transported into the cave from the area surrounding Sierra de Atapuerca. One of these two very large cores is the largest archaeological specimen from TD6 (270x150x100 mm) (see Fig 23B). It was worked using trifacial orthogonal knapping, and two big flakes refit with it, as detailed below. Therefore, during the TD6.2.0–1 occupation, hominins continued the practice of transporting large fragments of Neogene chert into the cave, although these blocks were underexploited.

The group of flakes (Fig 21) comprises 107 items (51.4%) made from all the raw materials, although mainly Neogene chert (31%), Cretaceous chert (18%) and quartzite (16%). The percentage of fracturing is slightly reduced (20%) compared with the preceding layer, but fluvial materials are still present in higher proportions.

There are 19 flakes smaller than 20 mm and 5 larger than 60 mm. Only one flake exceeds 100 mm (165x170x70 mm). After being detached, this large flake was knapped as a core. The average size of the complete flakes is slightly smaller than in preceding layers (30x28x10 mm), but their technical features are similar. They may have one to three dorsal scars, and are mainly non-cortical or with small cortical areas. Their butts are flat, either natural or unifaceted. The ventral delineation is varied and the bulbs are diffuse. The flaking angle ranges between 85° and 135°. The morphologies vary greatly, but trapezium shapes dominates. Particularly noteworthy items include fluvial materials with long, sharp edges.

The number of retouched tools documented increases in this group of layers (10%) (Fig 22). One pebble tool made of sandstone has been recovered, interestingly the only chopper from TD6 (Fig 22A). There are also 20 retouched flakes made from all the all raw materials, but primarily from Cretaceous chert (32%). The average size of the retouched flakes is clearly greater than the simple flakes (45x41x20 mm). This suggests, therefore, that larger flakes were intentionally selected to be retouched. However, retouched flakes do not differ from simple flakes in their technical features. The amount of edge retouching is slightly greater, going from a quarter of the perimeter to one half. Retouching is semi-abrupt or simple, denticulate and varied in delineation. One piece is not preserved well enough to allow its adscription, but the other tools are three side-scrapers, four notches, none denticulate scrapers (two carinated), and three denticulate points, two of which are typologically Tayac points (Fig 22H).

TD6.2.0–1 contains two groups of refits (see the Refits section) (Fig 23). The first is made up of one broken flake and one retouched quartzarenite flake, on which the fracture occurred during the process of either detaching or retouching the flake (Fig 23A). The second refit is made up of three Neogene chert pieces: two flakes and the largest core found in TD6. These belong to a knapping sequence, but the flakes are from different faces of the core (Fig 23B).

### TD6.1

**Fig 24:**
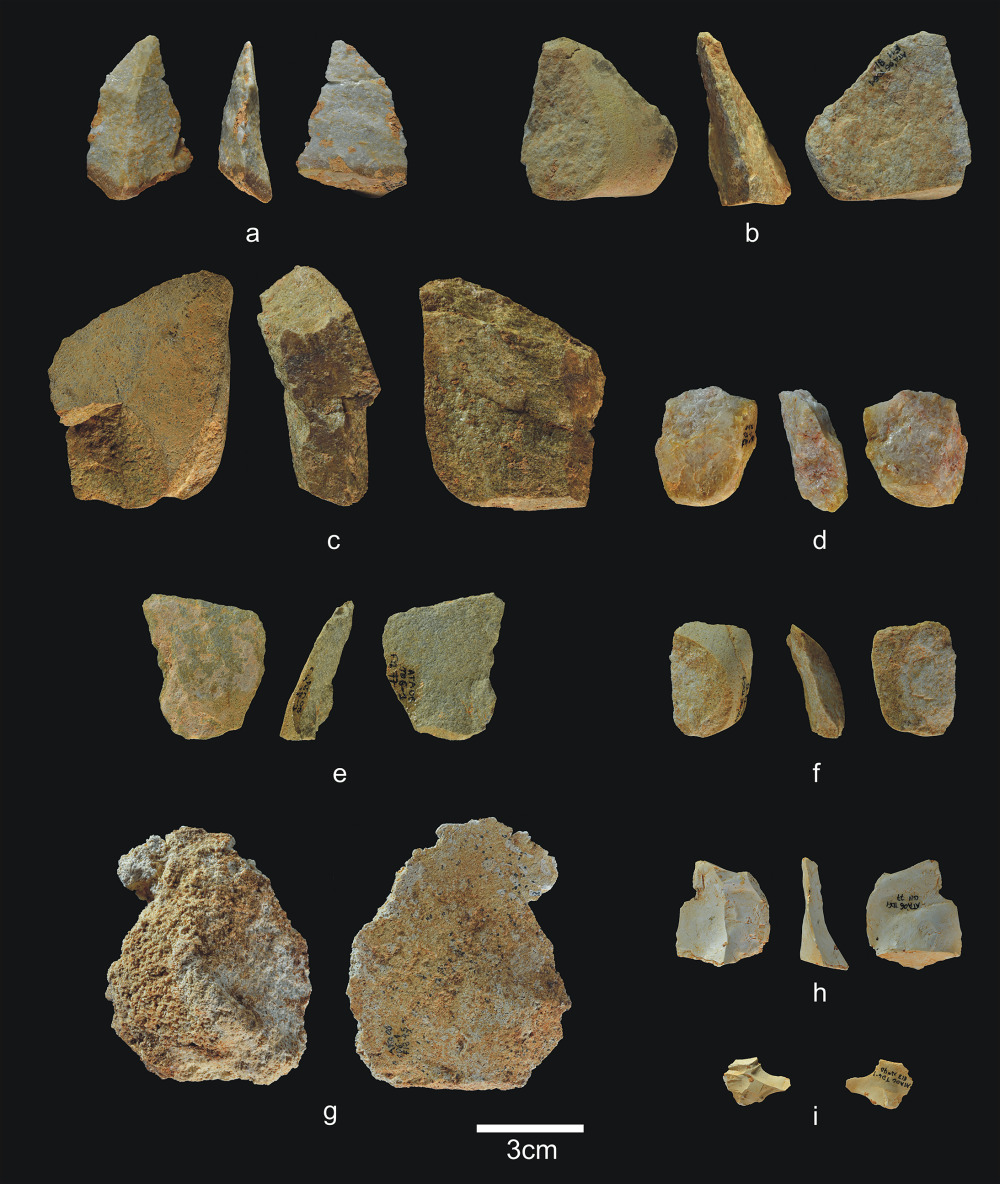
Simple flakes from TD6.1. a) Ata06-F11-48, quartzite; b) Ata06-F11-91, quartzarenite; c) Ata06-F13-118, quartzarenite; d) Ata06-F11-90, quartz; e) Ata06-F12-77, sandstone; f) Ata06-G11-8, sandstone; g) Ata06-F12-45, Neogene chert; h) Ata06-G11-77, Neogene chert; i) Ata06-E13-40, Cretaceous chert.

**Fig 25:**
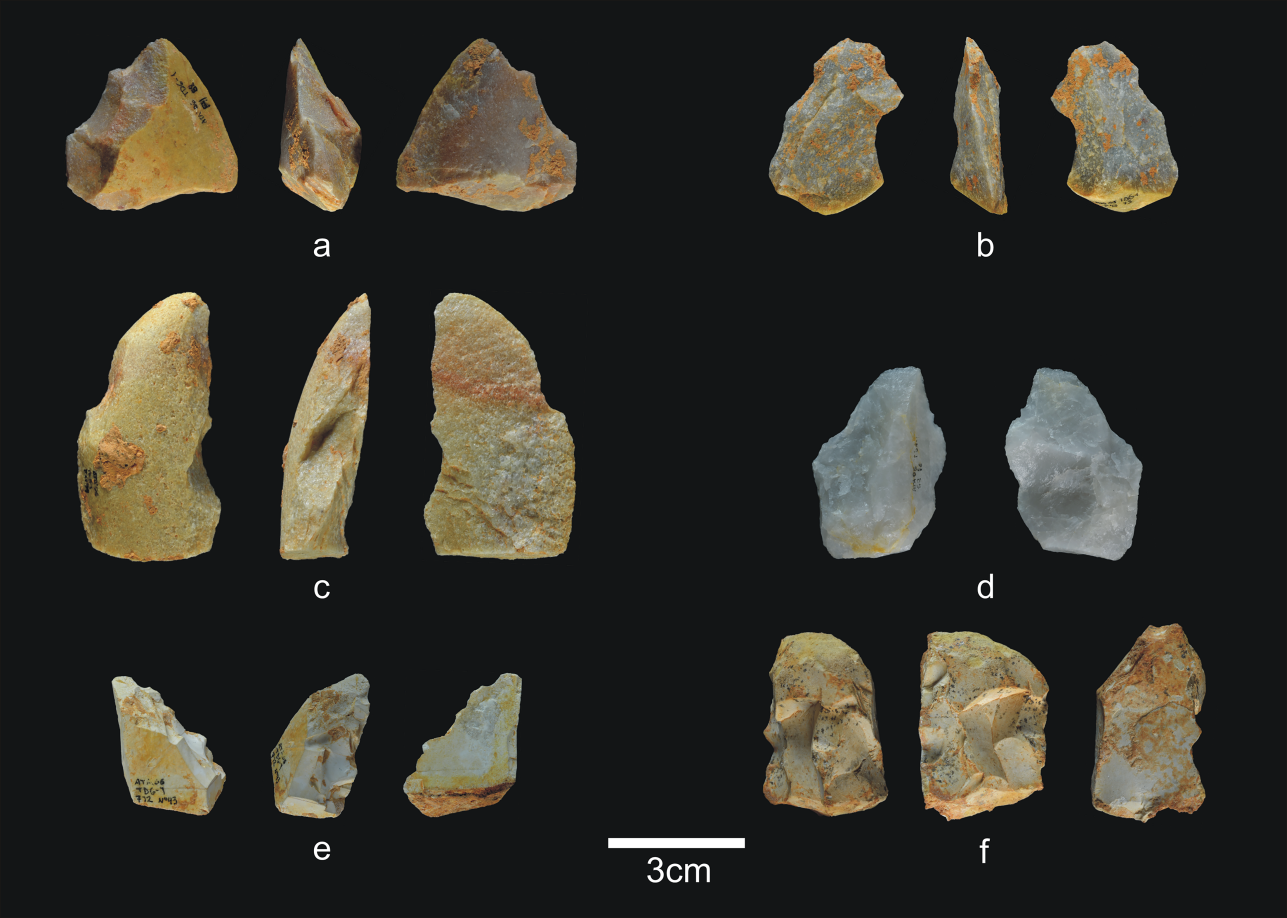
Retouched flakes from TD6.1. a) Ata06-F11-88, épine, quartzite; b) Ata06-F13-73, denticulate sidescraper, quartzite; c) Ata06-F11-99, denticulate sidescraper, quartzite; d) Ata06-G09-16, épine, quartz; e) Ata06-F12-43, denticulate carinated sidescraper, Cretaceous chert; f) Ata06-G09-15, denticulate carinated sidescraper, Cretaceous chert.

This is the final deposit in TD6, formed by channel and floodplain *facies* [1]. It has only been distinguished in the central area of the site, and contains 822 faunal remains (13 antler fragments, 228 coprolites, 29 teeth, and 552 bones), six hominin remains (one tooth and five bones), and 124 lithic items (Table 7) (Figs 24 and 25). Hominin 8 (*c*. 4 years old) belongs to this layer, as does the tooth of an immature individual. An adult individual has also been identified. This layer is characterised by the presence of 228 coprolites, leading to its interpretation as hyena latrine [73].

**Table 7:** Number and type of lithic items at TD6.1.

TD6.1	Sand	Lime	Qrtz	Qz	Qrtza	Chert	Cret	Neog	Total
**Retouched flake**		1	4	2			2	5	**14**
**Flake**	2		3	3	6		11	14	**39**
**Fractured flake**	2				2		2	2	**8**
**Fragment of flake**	1		2	1	1		2	1	**8**
**Fragment/chunk**	1	3			2		1	1	**8**
**Indet.**	3					1	2	41	**47**
**Total**	**9**	**4**	**9**	**6**	**11**	**1**	**20**	**64**	**124**

Indet: indeterminable pieces due to their poor preservation. Sand: sandstone; Lime: limestone; Qrtz: quartzite; Qz: Quartz; Qrtza: quartzarenite; Chert: indeterminate chert; Cret: Cretaceous chert; Neog: Neogene chert.

More than 35% of the lithic assemblage comprises indeterminable pieces of Neogene chert. In addition to sedimentary reasons, this poor state of preservation could be related to the chemical alterations produced in a hyena latrine [1].

Unlike the other layers, TD6.1 contains no pebbles or cores. Retouched flakes and simple flakes have been documented in all the raw materials, but predominantly in Neogene and Cretaceous chert. The percentage of fracturing in the flakes is high at 29%, but for the first time this figure is similar for all the raw materials.

There are 10 flakes smaller than 20 mm and three larger than 60 mm, but none reaches 70 mm (Fig 24). The average size of the complete flakes is the smallest in TD6 (28x27x11 mm), but again, the technical features are the same as those found in other layers. They are mainly non-cortical or have a small cortical area on their dorsal surfaces, with flat butts that are either cortical or unifaceted. Ventral delineations are varied and bulbs are equally marked and diffuse. The flaking angles range between 75° and 140°, and the morphologies are diverse, with a predominance of trapeziums.

The 14 retouched flakes (11%) (Fig 25) are on average larger than the simple flakes (35x26x14 mm), pointing to differential selection of larger flakes for retouching. Flake-blanks from retouched flakes and simple flakes are technically indistinguishable. The retouching of the edges is limited to 25% of the perimeter, is semi-abrupt or simple, invasive on the edge and the surface, produced from the ventral face in most cases, denticulate and with frequent sinuous delineation. Thirteen items are suitable for analysis and these are: one marginal abrupt retouched flake, one notch, three *épines*, seven denticulate side-scrapers and one denticulate point.

TD6.1 is the only layer in which no clear refits have been identified.

## Refits

**Fig 26:**
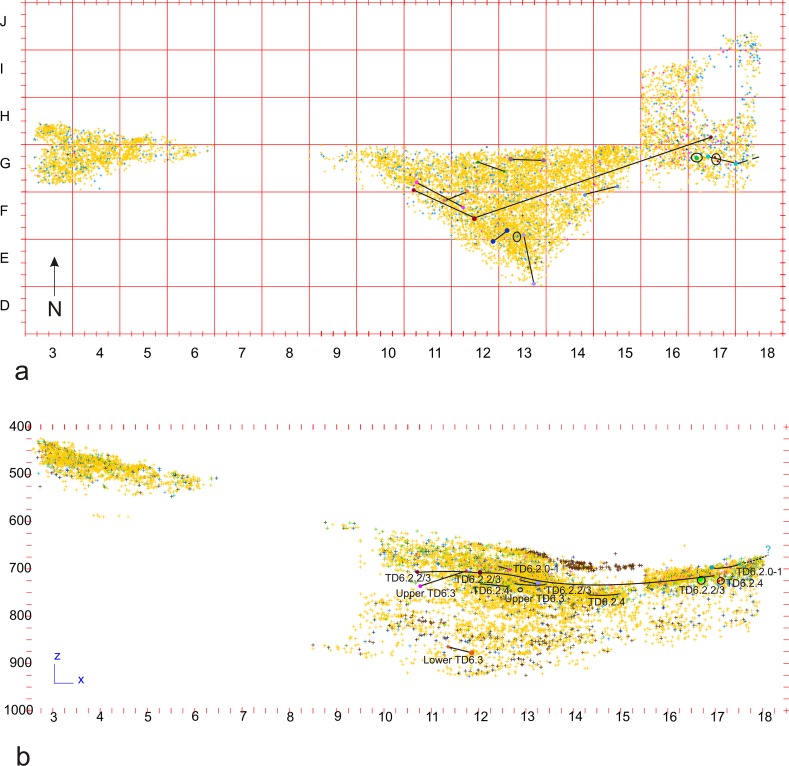
Lithic refits of TD6. Surface (a) and vertical (b) views of the TD6 lithic refits together with the distribution of the entire faunal (except coprolites) and lithic assemblage.

Table 8 shows all of the lithic refits found in unit TD6. There are 12 groups of refits comprising 26 pieces, which is a high proportion considering that the excavated area is about 20m^2^ and is at the side of the cave (see Fig 1). Refits of all raw materials have been found with the exception of Cretaceous chert. They have been found in all layers of TD6 (Fig 26), with the possible exception of TD6.1, where there is a problematic Neogene chert refit involving one broken flake and a flake fragment, both of which are highly altered and poorly preserved, in such a way that it is difficult to distinguish whether the fracture is recent or not. This refit has not been considered in this work.

**Table 8:** Lithic refits from TD6.

	RAW MATERIAL	YEAR	SUBUNIT/LAYER (this work)	LAYER from EXCAVATION	SQUARE	No.	PIECE	DISTANCE BETWEEN PIECES	TYPE OF REFIT
1	Quartzite	2010	Lower TD6.3	TD6.3.6	F11	97	Fragment of pebble	45 cm	Fracture of hammerstone
		2010	Lower TD6.3	TD6.3.6	G12	293	Hammerstone		
2	Quartz	2008	Upper TD6.3	TD6.3.1	F13	9	Core	5 cm	Knapping process, possibly bipolar on anvil
		2008	Upper TD6.3	TD6.3.1	F13	10	Flake fragment		
3	Quartzarenite	2008	Upper TD6.3	TD6.2.4	F12	1	Broken flake	110 cm	Knapping process
		2009	Upper TD6.3	TD6.3.1	G11	15	Core		
4	Limestone	1995	TD6.2.4	42	G17	249	Flake	10 cm approx.	Knapping process
		1995	TD6.2.4	42	G17	243	Retouched flake		
5	Quartz	2004	TD6.2.4	TD6.2.4	G15	22	Flake	60 cm	Knapping process
		2005	TD6.2.4	TD6.2.4	F14	4	Flake		
6	Quartzarenite	2009	TD6.2.4	TD6.2.4	G12	374	Broken flake	55 cm	Siret fracture
		2009	TD6.2.4	TD6.2.4	G13	562	Flake fragment		
7	Sandstone	1995	TD6.2.2/3	40–41	H17	137	Flake	768 cm	Knapping process
		2003	TD6.2.2/3	TD6.2.2/3	G11	1	Flake		
		2007	TD6.2.2/3	TD6.2.2/3	F12	147	Core		
8	Ortho-quartzite	2007	TD6.2.2/3	TD6.2.2/3	E13	130	Core	100 cm	Fracture during knapping
		2007	TD6.2.2/3	TD6.2.2/3	F13	395	Flake fragment		
9	Quartzarenite	2009	TD6.2.2/3	TD6.2.2/3	G13	383	Broken flake	60 cm	Distal fracture, unknown origin
		2009	TD6.2.2/3	TD6.2.2/3	G13	384	Flake fragment		
10	Neogene chert	1995	TD6.2.2/3	40–41	G17	166	Flake	< 5 cm	Fracture at knapping?
		1995	TD6.2.2/3	40–41	G17	169	Flake		
11	Quartzarenite	2007	TD6.2.0–1	TD6.2.0	E12	4	Broken flake	25 cm	Possible fracture during knapping or retouching
		2007	TD6.2.0–1	TD6.2.0	F13	144	Retouched flake		
12	Neogene chert	1986	TD6.2.0–1	*	S/C	1	Core	c. 70 cm between flakes*	Knapping process
		1994	TD6.2.0–1	38	G17	20	Flake		
		1994	TD6.2.0–1	37	G18	1	Flake		

*: Unfortunately, the core was recovered out of context in 1986

The types of refits represented in TD6 are conjoins between pieces fractured during knapping or retouching (i.e. Siret and other accidental fractures), and refits resulting from the core knapping process. None of the refits involves more than three pieces.

The most spatially distant pieces belong to a sandstone refit from layer TD6.2.2/3 in the eastern area that correlates with layer 40–41 from the central excavation. This act makes this particular refit very important in terms of archaeostratigraphic correlation. It comprises three pieces (see Fig 18A): one core and two flakes. One of the flakes (30x19x8 mm) belongs to a series of similar removals made on a pebble. It was found in the eastern sector of the excavated surface. The other flake is medium sized (65x85x23 mm), and bears the scars of the abovementioned removals. It was found approximately one metre away from the pebble core, which was abandoned with no further flaking. These two pieces were recovered at a distance of approximately seven metres from the first small flake.

In addition to this important refit, other refits also provide significant insight into actions carried out inside the cave. This is the case of fractures (conjoins) whose pieces are separated 5 cm, 25 cm, 50 cm, and as much as 100 cm from one another, which are proven distances for *in situ* knapping accidents [74, 75]. There are also refits pertaining to knapping processes, the closest two pieces being 5 cm apart, and the most distant separated by 110 cm.

No refit have been documented between pieces from different, non-correlating layers. The only exception is refit code CZT-1-TD6 (see Table 8), formed by one broken quartzite flake assigned during the excavation to layer TD6.2.4 and a core assigned to layer TD6.3.1, which is immediately underlying TD6.2.4. However, the small area of TD6.2.4 where the flake was embedded is a sedimentary wedge, similar to the underlying subunit TD6.3.1, and different from the general TD6.2.4 deposit. It therefore seems that the flake was wrongly assigned to TD6.2.4 during the excavation, and we decided to ascribe this refit to the Upper-TD6.3 stage, where layer TD6.3.1 has been ascribed in this work.

No particular concentrations of and/or gaps in raw materials or categories of pieces have been identified. However, and given that this work is based on a 20m^2^ area at the edge of the occupation, spatial distribution analyses will only provide reliable data when the entire surface of TD6 has been excavated.

Finally, it is interesting to note that the connections between the refit pieces show no specific pattern of orientation. Furthermore, some of the refits in TD6.2 made up of pieces found in close proximity to one another correspond to knapping sequences, and are located in the southern area of the excavation (see Fig 26). Taken together, these data seem to contradict the interpretation of Campaña and colleagues [1] regarding the existence of two different influxes of sediments and archaeo-paleontological remains in TD6.2, one in the northwest of the cave, and the other in the south of the cave.

## Discussion

This work aimed to update the information available on the lithic assemblage found in Gran Dolina unit TD6, now that the latest excavations in the southern section of the site have been completed, and to identify changes in the occupational patterns and technological strategies of the hominins that occupied the cave. In fact, this is the first study to analyse the three subunits of TD6 (TD6.1, TD6.2 and TD6.3), as only data on TD6.2, and recently TD6.3 [72] has been published to date.

The lithic assemblage of TD6 can be divided into two groups: that belonging to the underlying subunit TD6.3 and the remainder of TD6 (TD6.2 and TD6.1). This distinction is based on the technological composition of the subunits: TD6.3 has a poor lithic assemblage, mainly made up of pebbles, most of which present percussion marks and chipping, and which we have grouped as “percussive material”. This evidence is particularly clear in the oldest layers (Lower-TD6.3), as technological diversity increases in younger subunit (Upper-TD6.3). In addition, these evidences are coincident with the zooarchaeological interpretation of this sub-unit, where hominins were merely marginal and sporadic visitors [72]. In contrast, the lithic assemblages of TD6.2 and TD6.1 contain a wealth of items from various categories in which production processes were habitual. Seven varieties of raw materials were used; some were differentially selected for particular tools, such as retouched flakes made from Cretaceous chert [62, 63]. Knapping was common, as seen by the number of cores and their products, but evidence of percussion activities is always notably present. Indeed, most of the complete and fractured pebbles, from all the layers, bear percussion marks. Although there was no morphological selection of the pebbles, half of the hammerstones of TD6 were quartzite, which is one of the hardest materials present in Atapuerca.

Three main knapping methods were employed: unipolar longitudinal, multifacial orthogonal and centripetal, in addition to the bipolar-on-anvil method, which is almost entirely restricted to quartz cores. Whatever the raw material, cores were abandoned at all stages of flaking in all the layers, although half of those abandoned in their initial stages belong to TD6.2.2/3, half of the cores abandoned at an intermediate stage of production were documented in layers TD6.2.4 and TD6.2.0–1, and half of the exhausted cores were abandoned in TD6.2.0–1. These results would appear to have an occupational significance, although they are difficult to interpret before the excavation of TD6 is entirely completed.

The flakes detached from these cores are not standardised, as their technical features vary greatly. The retouched tools are mainly the denticulate type, including notches, *épines*, denticulate scrapers, and points, with some notable Tayac Points. The flake-blanks for retouching show the same technical features as simple flakes, but are a little larger in most layers.

Several noteworthy observations have arisen with regard to the raw materials in the unit. Interestingly, the less significant the hominin occupation (TD6.3) [72], and consequently the lower number of items, the greater the proportion of fluvial materials, probably related to percussive tasks during the occupation. As soon as the hominin presence in the cave became more pronounced (TD6.2), the diversity of raw materials increased, as did the variety in the lithic assemblage. However, from this time onwards, Neogene chert was always the dominant raw material.

Core knapping presents one of the strongest pieces of evidence for differential raw material usage: Fluvial cobbles and pebbles were mostly knapped using the unipolar longitudinal method, although this technique was also applied to chert. On the other hand, centripetal and multifacial orthogonal knapping were principally used with Neogene and Cretaceous chert. In addition, the bipolar-on-anvil technique was mostly used to work quartz pebbles. Taken together, it seems that two flaking methods were the background and dominant techniques for the TD6 hominin community: 1) the unipolar longitudinal knapping method, as applied to all materials; and 2) multifacial orthogonal knapping, for Neogene and some Cretaceous chert. The hominins selected large, angular, massive cobbles of fluvial materials for the former; and big blanks of Neogene chert, as well as some small irregular nodules of Cretaceous chert, for the latter.

It is possible that these long-term knapping strategies began as technical responses in case that hominins were not skilled enough to control the original volumes through knapping. This way, the hominins appear to have adapted to the original volumes, instead of changed them. In the long term, this technical practice may have given rise to a habitual behaviour even when flat blanks were available. Flat blanks are ultimately bifacial blanks, and are suitable for knapping methods such as the centripetal technique. Flat blanks near the Sierra de Atapuerca can be found as flat pebbles, as well as large flakes of Neogene chert that could have been produced when trying to extract fragments from the huge blocks scattered around the Sierra. However, the hominins from Gran Dolina either did not produce these or did not choose to transport them into the cave. In addition, they usually selected quadrangular and irregular pebbles for flaking. It is possible that bifacial blanks were of little use with the technical strategies they employed, and therefore they did not use them. In short, the bifacial management of blanks is absent.

However, they eventually began to develop the centripetal knapping trend, and they undertook this mainly on small, irregular Cretaceous chert nodules. These nodules are not especially flat, but they are small, so the likelihood of creating an adequately angled percussion platform with the first detached flakes is greater than when using larger Neogene chert fragments. Cretaceous chert is higher quality than Neogene chert, and the percussion platform and the small size of the nodules means the flakes encompass the entire thickness of the core. Therefore, the easiest way to knap these nodules is by making an initial strike, then striking a place adjacent to the site of the first strike, and then striking a place adjacent to that second site, and so on. This eventually leads to centripetal knapping. The high quality of the Cretaceous chert may also be the reason it was differentially selected for small retouched tools.

Interestingly, all these characteristics are maintained throughout the stratigraphy. TD6.2 and TD6.1 evidence a very homogeneous lithic industry in terms of both technical and raw material usage, with the exception of few features that seem to be related to occupational traits. This may explain the increased production of retouched flakes over time, and the presence of large Neogene chert cores in some layers of subunit TD6.2. The presence of these large cores with little knapping denotes the fact that these occupations were planned, and that the hominin impact was greater, since they thought they would require such large quantities of chert. It also coincides with episodes of more substantial and more frequent cannibalism.

Furthermore, the fact that these large cores are distributed throughout the excavated areas of TD6.2.2/3 and TD6.2.0–1 supports the argument for the limited diachronous occurrence of these occupation events.

Our results show no evidence of technological evolution, merely an increase in lithic production up through the stratigraphy, and certain features that may point to occupational differences. Technically, the TD6 lithic assemblage is very homogeneous; this may be because the hominin occupations occurred more or less consecutively over quite a short period. However, the long-lasting technical character of European Mode 1 technology makes finding evidence of change unlikely. Nevertheless, some elements must be taken into account: 1) the existence of technological evidence pointing to planned occupations; 2) the use-wear identified on a TD6 tool sample related to butchering processes, where evidence for cutting actions on soft animal tissues are well represented [58, 76]; 3) the presence of a MNI of 11 hominins whose carcasses were processed similarly [33, 56, 77, 78]; and 4) the butchering of other animal carcasses, through which researchers have identified hunting activities, carcass transport, social cooperation, and food sharing [56]. All this seems to point to repeated hominin occupations employing the same subsistence strategies in a context of complex social organisation. In addition, the presence of 12 groups of refits, extracted from almost all the layers in the TD6 stratigraphy, while the refits themselves are from single layers, is important evidence supporting the individualisation of the diachronically deposited hominin occupations. Taken together, the type, location, and distances between the TD6 refit pieces seem to indicate that they were produced *in situ*, and that any sediment movement, if present, was not significant. Interestingly, these results clearly contrast with recently published work, which claims a secondary position for TD6 [1].

Concerning the technological position that TD6 occupies with respect to other Early Pleistocene sites, its lithic industry seems to represent Mode 1 technology with archaic and derived features. Archaic features include both the use of a single flake-production method (usually unipolar longitudinal) and a diversification of flaking strategies such as orthogonal, bipolar-on-anvil, and centripetal, where none predominates. Examples of this can be seen in the deposits from Atapuerca-TE9 (1.2 Ma) [79, 80], Atapuerca-TD3-4 (c. 1 Ma) [63, 81], Le Vallonnet (France) (1,2–1,1 Ma) [82, 83], Monte Poggiolo (Italy) (c. 850 ky) [84, 85], and Pakefield (England) (c. 700 ky) [86]. Diversification of flaking strategies can be seen at sites in Atapuerca (TD6), Barranco León and Fuente Nueva (Spain) (1.3 Ma and 1.2 Ma respectively) [87, 88], and possibly Pirro Nord (Italy) (1.6–1.3 Ma) [84]. The bipolar-on-anvil technique has also been documented in TD6, at Fuente Nueva 3, and Barranco León, Pont de Lavaud (France) (1.1 Ma) [89, 90], and Vallparadís (Spain) [91] (990–780 ky) [92], although in Western Europe this strategy was frequently linked to raw material conditioning.

In contrast, derived features include the diversification of raw materials [71], the production of small retouched tools, the diversity of these tools, and their certain standardisation, regarding other European Mode 1 sites [62]. Some of the abovementioned sites yielded small retouched tools, although in small quantities (i.e. Barranco León, Pont de Lavaud, Le Vallonnet, Vallparadís, Pakefield, and Happisburgh (990–780 ky [93]), the last site without presence of cores. However, only TD6 contains seven raw materials used to produce a relatively large number of retouched flakes. This also reflects a certain degree of diversification and standardisation, although it only involves the sizes of the flakes for retouching, their thickness, and the way the denticulation was produced. The presence or absence of choppers and chopping tools seems not to have any particular technological meaning, as proposed elsewhere [62].

In our view, TD6 represents evolution and diversification within the European Mode 1, whose earliest representative is the lithic assemblage of Dmanisi (Georgia) [94, 95]. Although raw material differences may distance these lithic assemblages, both sites contain similar archaic technical features, such as significant use of pebbles for percussion activities, as well as derived technical features, like the diversity of knapping methods and a considerable potential for producing pieces of all types and morphologies [62, 63]. This is probably because they contain orthogonal core morphologies as well as bifacial ones, such as those produced by the centripetal method. This production potential includes a few large flakes that could have been shaped into large cutting tools, even though in TD6 they were ultimately used as cores, employed as they were for some use, or discarded. However, TD6 is younger than Dmanisi, so it would be logical to see more evolved behaviour here, representing a much later stage in the diversification of archaic technology [96]. As a result, TD6 contains a high percentage of small retouched flakes, which are lacking at Dmanisi and are somewhat unusual in the European Early Pleistocene.

An interesting question is whether the technology represented in TD6 might have evolved into the Acheulean industry, as it is known in Europe, such as the contemporary site of Barranc de la Boella (Tarragona) [97–99], and particularly other Sierra de Atapuerca sites, such as Galería [100, 101]. Although there is a gap in the hominin presence at Atapuerca, as well as in most of Europe during the Early to Middle Pleistocene transition [62, 102], an assemblage with the TD6 technical characteristics theoretically might have led into the European Acheulean *if* it acquired over time some features [62, 63]. These are: 1) the acquisition of the bifacial concept to use larger flakes to shape large bifacial tools, and/or diverting orthogonal morphologies to shape picks and other large non-bifacial tools; 2) the increase in the use of centripetal knapping as the dominant production method, maintaining some cores with unipolar longitudinal knapping; 3) the decrease in the use of the multifacial orthogonal knapping; 4) the production of thinner simple and retouched flakes; 5) the increase of extensive shaping in tool production; and 6) the increase of standardisation in the knapping products, including more flakes with their detaching axes parallel to the removals made on their dorsal surfaces. In the end, all these features are dependent on just a few technological characteristics: control of the original morphologies and raw materials volumes, instead of adapting to them; control of the bifacial concept for both flake production and tool shaping; and the standardisation of sizes, morphologies and retouch types. All these features were widely developed during the Acheulean.

## Conclusions

The TD6 archaeo-palaeontological record seems to represent two main stages of occupation. During the oldest (TD6.3), both the type of pieces and their scarcity evidence light and infrequent hominin occupation of the cave. This may represent a certain degree of continuity with the earliest hominin evidence at the site, from the lowermost units of Gran Dolina (TD3-4 and TD5). In fact, a great deal of evidence of carnivores and hyenas has been found in TD6.3, while the hominin impact is relatively scarce and marginal [72]. However, this does not take away from the fact that the hominin presence in the TD6.3 sequence becomes more significant and prolific in terms of tool production than the older layers, perhaps reflecting the establishment of the Gran Dolina cave as a landmark in the area, as evidenced by the TD6.2 and TD6.1 occupations.

The lithic assemblages from both TD6.2 and TD6.1 display a greater range of production processes, with a greater diversity of the different raw materials being used. These comprise a very rich and varied lithic assemblage associated with the processing of faunal carcasses and hominin bodies [33, 56, 57, 77]. Although the hominin presence increased over the time interval represented by TD6, technically the lithic assemblage is very homogeneous throughout the stratigraphy: cores were knapped in the same way, and knapping products and tools have similar features. This diachronous technological homogeneity, together with the presence of certain features that may be related to occupational strategies (i.e. cannibalism), might indicate that the time between TD6.2 and TD6.1 was short, and probably reflects the activities of hominin communities that repeated the same subsistence decisions. The presence of 12 groups of lithic refits at various points throughout the entire TD6 strata, most of which are made up of pieces found in close proximity to one another, is evidence of the good preservation of the site and the *in situ* character of the various hominin occupations; this also raises doubts about recent interpretations of the TD6 unit as being in a secondary position [1].
